# Comparison of targeting two antigens (GPA33 versus HER2) for ^225^Ac-pretargeted alpha-radioimmunotherapy of colorectal cancer

**DOI:** 10.7150/thno.116062

**Published:** 2025-06-20

**Authors:** Sara S. Rinne, Daniela Burnes Vargas, Brett A. Vaughn, Sumudu Katugampola, Brian W. Miller, Darren R. Veach, Blesida Punzalan, Elisa de Stanchina, Rona Yaeger, Ileana C. Miranda, Hong Xu, Hongfen Guo, Jazmin Schwartz, Edward K. Fung, Roger W. Howell, Steven M. Larson, Nai-Kong V. Cheung, Sarah M. Cheal

**Affiliations:** 1 Department of Radiology, Weill Cornell Medicine, New York, NY, USA.; 2 Department of Pediatrics, Memorial Sloan Kettering Cancer Center (MSKCC), New York, NY, USA; 3 Molecular Pharmacology Program, MSKCC, New York, NY, USA.; 4 Division of Radiation Research, Department of Radiology and Center for Cell Signaling, New Jersey Medical School, Rutgers University, Newark, NJ, USA.; 5 Department of Radiation Oncology, Department of Medical Imaging, College of Medicine, University of Arizona, Tucson, AZ, USA.; 6 Department of Radiology, MSKCC, New York, NY, USA.; 7 Antitumor Assessment Core Facility, MSKCC, New York, NY, USA.; 8 Department of Medicine, MSKCC, New York, NY, USA.; 9 Laboratory of Comparative Pathology, Weill Cornell Medicine, MSKCC, The Rockefeller University, New York, NY, USA.; 10 Department of Medical Physics, MSKCC, New York, NY, USA.

**Keywords:** targeted alpha therapy, Actinium-225, glycoprotein A33, human epidermal growth factor receptor 2, pretargeted radioimmunotherapy

## Abstract

**Purpose:** Curative therapies remain a significant unmet need for advanced human colorectal cancer (CRC). The aim of this study was to establish a 3-step ^225^Ac-DOTA pretargeted radioimmunotherapy (PRIT) system for human CRC, targeting two individual antigens: glycoprotein A33 (GPA33) and human epidermal growth factor receptor 2 (HER2).

**Methods:** *In vitro* cellular uptake and internalization assays, as well as survival assays (colony forming) were performed in GPA33- and HER2-positive SW1222 human CRC cells. *In vivo* biodistribution and therapy studies were performed with two human CRC xenograft mouse models.

**Results:** For both antigens, treatment with up to 74 kBq ^225^Ac-DOTA-PRIT in SW1222-tumored mice significantly enhanced overall survival in comparison to controls, including histologic cures. The uptake of ^225^Ac at the tumor correlated with antigen expression (antigen expression for GPA33:HER2 is 5:1). Cellular assays showed no significant differences in internalized fraction or nucleus-associated radioactivity between the two targets. GPA33 had a higher absorbed dose to the nucleus (1.3 Gy *vs.* 0.65 Gy for HER2), resulting in reduced clonogenic survival. A single cycle of either GPA33 or HER2 DOTA-PRIT (37 kBq; 193.31 Gy and 113.91 Gy (relative biological effectiveness [RBE] = 5), respectively) was equally effective. No chronic nephrotoxicity was seen at ≤ 20 Gy (RBE = 5). The efficacy of GPA33-directed ^225^Ac-DOTA-PRIT was also confirmed in a patient-derived xenograft model.

**Conclusion:** In summary, ^225^Ac-DOTA-PRIT to GPA33 or HER2 was highly effective and safe in preclinical models of human CRC. Tumor response to treatment could not be predicted by nuclear absorbed dose alone, highlighting the importance of comprehensive micro- and macro-dosimetry.

## Introduction

Colorectal cancer (CRC) is a significant global health concern, accounting for about 10% of cancer cases worldwide, according to the WHO. Treatment options depend on factors such as cancer stage and patient health and can include surgery, chemotherapy, radiation therapy, and targeted therapy, often in combination. Although cures are possible with localized disease, durable control of metastatic CRC remains an urgent currently unmet challenge 1. Radioimmunotherapy (RIT) offers an alternative approach, using antibodies to deliver a radioactive, cytotoxic payload specifically to tumor cells. This method is advantageous as it can target both macroscopic tumors and micrometastases while minimizing radiation exposure to healthy tissue.

The radiation doses necessary for tumor ablation are limited by the absorbed doses to healthy tissue. One of the major hurdles in RIT is the long circulatory half-life of antibodies leading to unintended myelotoxicity. Radiolabeled peptides with much faster pharmacokinetics for peptide receptor radionuclide therapy (PRRT) can reduce the radiotoxicity to blood. However, renal trapping of peptides in the proximal tubules of the kidney after glomerular filtration leads to an increased risk for nephrotoxicity with PRRT 2. Pretargeted radioimmunotherapy (PRIT) can circumvent the limitations of traditional RIT and PRRT by separating the antigen binding from the radioactive payload delivery in a multistep approach. In the first step, a bispecific antibody (BsAb) with specificities for the tumor and for the payload is allowed to bind to the tumor. With time, unbound BsAb is cleared from circulation or actively removed by a clearing agent (CA). In the last step, the radioactive payload is delivered in the form of a small molecule with rapid tumor penetration and renal clearance, achieving exceptional therapeutic indices (TIs; tumor-to-normal tissue-absorbed dose ratios) for RIT. Several recently published reviews provide excellent overviews of different PRIT approaches and illustrate the modularity of PRIT in targeting a wide range of tumor antigens with diverse payloads 3-5.

From a radiobiological perspective, ⍺-emitters such as ^225^Ac, ^211^At, ^212^Bi, or ^212^Pb are extremely potent payloads for targeted radiotherapy, including PRIT. Due to their high linear energy transfer, they create dense and complex ionizations along their path, resulting in DNA double-strand breaks and damage estimated to be several folds more effective than the damage caused by β-emitters and external beam radiation. Recently, we have adapted a BsAb/DOTA-radiometal-based PRIT system (DOTA-PRIT) for targeted ⍺-therapy (TAT) with ^225^Ac 6. This PRIT approach uses a BsAb in the IgG-scFv format (210 kDa) 7, which contains sequences for an anti-tumor antigen IgG and an anti-DOTA(metal) scFv. The anti-DOTA(metal) scFv is an affinity-matured version of the anti-DOTA(metal) antibody 2D12.5, with affinities of approximately 10-15 picomolar for lutetium or yttrium complexes 8. To achieve high TIs, we use a CA (either DOTA-dendron 9 or DOTA-dextran 10) to reduce circulating IgG-scFv prior to injection of the DOTA-radioligand. A typical regimen consists of three steps: administration of BsAb at *t* = -28 h, followed with administration of CA (*t* = -4 h) and radioligand (*t* = 0 h). Initial therapy experiments using a specialized carrier for ^225^Ac ([^225^Ac]Ac-Proteus-DOTA or [^225^Ac]Ac-Pr) demonstrated complete responses and histological cures in subcutaneous breast cancer, CRC, and neuroblastoma xenografts, as well as an ovarian peritoneal carcinomatosis model, with only mild toxicity 6, 11.

Most RIT approaches against CRC have been based on targeting the human carcinoembryonic antigen using IgG antibodies, for example, ^90^Y-M5A and ^131^I-labetuzumab 12, 13. Two other molecular markers holding promise for RIT of CRC are glycoprotein A33 (GPA33) and human epidermal growth factor receptor 2 (HER2). GPA33 is expressed in 95% of CRC patients 14. Because of its limited expression in healthy tissue (colon and bowel epithelium), it has been extensively studied for RIT of CRC, which our team and others have previously demonstrated 15, 16. Clinical studies of GPA33-immunoPET with nanobody-based probes are ongoing (NCT06203587). HER2 is an emerging target in metastatic CRC 17. Although high levels of HER2 overexpression is found in only 6% of tumors 18, low HER2 density CRC appears to be relatively frequent 19. Despite the low expression of HER2, clinical trials have demonstrated the activity of dual HER2-targeted therapy with trastuzumab plus tucatinib, which is approved for HER2-positive metastatic CRC, and of the antibody-drug conjugate trastuzumab deruxtecan, which is approved for metastatic CRC with 3+ HER2 expression by immunohistochemistry (IHC).

In the present study we report high-TI ^225^Ac-DOTA-PRIT in a preclinical model of CRC targeting two different tumor-associated antigens: GPA33 and HER2. GPA33 is known to have 5- times as many binding sites on SW1222 CRC as HER2. Our hypothesis was that this difference would directly correlate with the relative effectiveness of the *in vivo* response and the curative potential. We hypothesized that targeting GPA33, the antigen with the greatest tumor expression and thus the most favorable tumor-absorbed dose, would lead to greater tumor control in comparison to HER2 during head-to-head studies. In a comprehensive approach including micro- and macro-dosimetry, *in vitro* and *in vivo* efficacy studies, and evaluation of in xenograft and PDX models study, we aimed to refine and better understand the underlying mechanisms contributing to the exceptional properties of ^225^Ac-DOTA-PRIT.

## Material and Methods

### General

The bispecific anti-GPA33/anti-DOTA (anti-huA33-C825 BsAb, 210 kDa) and anti-HER2/anti-DOTA (anti-HER2-C825 BsAb, 210 kDa) antibodies were produced as previously described 16, 20. Please see Table S1 for BsAb binding affinities to target antigens measured by surface plasmon resonance (Biacore T100). The DOTA-dendron CA CCA α-16-DOTA-Y^3+^ (MW: 9059 Da), consisting of a nonradioactive yttrium-DOTA-Bn molecule attached via a linker to a glycodendron displaying 16 terminal α-thio-N-acetylgalactosamine (α-SGalNAc) units, was prepared according to reported methods 9. Proteus-DOTA (Pr), the precursor to the ^111^In and ^225^Ac-labeled radioligand, consists of a three-arm DOTA radiometal-chelating agent (1,4,7,10-tetraazacyclododecane-1,4,7-triacetic acid; DO3A) separated by a tetraethylene glycol (PEG_4_) linker to a ^175^Lu (natural) lutetium complex of benzyl-DOTA (MW: 1350 Da) 6. All PRIT reagents were formulated in sterile isotonic saline solution and administered intravenously via the tail vein. The GPA33- and HER2- expressing human CRC cell lines SW1222 and LS174T were obtained from the Ludwig Institute for Cancer Immunotherapy (New York, NY) or purchased from ATCC (Manassas, VA, USA), respectively. SW1222 cells were maintained in IMDM media supplemented with 10% FBS, 2 mM glutamine, 100 units/mL penicillin, and 100 units/mL streptomycin. LS174T cells were maintained in RPMI1640 with 10% FBS, 2 mM glutamine, 100 units/mL penicillin, and 100 units/mL streptomycin. GPA33 and HER2 antigen density of SW1222 and LS174T cells was measured by fluorescent-activated cell sorting with Quantum™ Simply Cellular^®^ beads (Bangs Laboratories, Inc.) according to the manufacturer's instructions.

### Radiochemistry

Chemical synthesis of Pr and subsequent radiolabeling with [^225^Ac]Ac(NO_3_)_3_ yielding the [^225^Ac]Ac-Pr radioligand was performed according to previous methods 6.

For ^111^In-labeling of Pr, 4 nmol of Pr was added to 100 µL of 0.2 M ammonium acetate buffer (pH 5.3) in a polypropylene vial. [^111^In]InCl_3_ solution (7 µL, 92.5 MBq [2.50 mCi]), obtained from Nuclear Diagnostic Products, Rockaway, NJ, USA) was added to the vial and mixed gently by pipetting. The mixture was heated for 30 min at 90 °C and cooled. A small amount (2 µL) was removed to determine radiochemical yield (RCY) and radiochemical purity (RCP) by HPLC (>99% RCY and RCP). The product was used without further purification.

### GPA33 and HER2 antigen density

The GPA33 and HER2 antigen levels determined by fluorescent-activated cell sorting for SW1222 were an average of 151904 and 56765 sites/cell, respectively. For LS174T, the GPA33 antigen levels and HER2 antigen levels were an average of 216461 and 57582 sites/cell, respectively.

### *In vitro* uptake and internalization assays

The *in vitro* uptake and internalization kinetics of the anti-GPA33- and anti-HER2- pretargeted tracer radioligand were investigated. [^111^In]In-Pr was used as a surrogate for [^225^Ac]Ac-Pr 6. In brief, CRC cells (SW1222 or LS174T) were pre-incubated with 64 nM of anti-GPA33 or anti-HER2 BsAb for 1 h at 37 °C. Following removal of the antibody-containing media, cells were incubated with 22 nM [^111^In]In-Pr for up to 24 h. At selected timepoints, the membrane-bound activity, activity in the cytoplasm, and the activity in the nuclei were collected. Cells were first incubated with 0.2 M glycine buffer with 0.15 M NaCl and 4 M urea 21 to collect the membrane-bound activity. A nuclei isolation kit (Nuclei EZ Prep, Sigma Aldrich) was used according to the manufacturer's instructions to separate the nuclei from the cytoplasm. A more detailed description is provided in the supplementary material.

### In vitro cell survival experiments

SW1222 cells (500,000 cells/mL in 0.6 mL, in suspension in vented culture tubes) were incubated with 64 nM of BsAb for 1 h at 37 °C on a rocker-roller. Next, cells were washed twice with serum-free media. The cells were resuspended in 1 mL of serum-free medium with [^225^Ac]Ac-Pr (0-20 kBq/mL) and suspensions were placed back on the rocker roller for 90 min at 37 °C. As a final step, cells were washed twice with serum-free media, counted, and plated for colony formation at different densities. After 21 d, cells were fixed and stained using 6% glutaraldehyde with 0.5% crystal violet 22. Colonies (>50 cells) were counted, and surviving fractions were calculated. Additional details are in Figure S1 and the supplementary data.

### Multicellular dosimetry

Cellular-level dosimetry of the SW1222 cells was performed using the multicellular dosimetry software tool, MIRDcell V4.14 23-25. For dosimetry purposes, within MIRDcell, SW1222 cells were modeled with a concentric spherical geometry with 6 μm as a cellular radius and 4 μm as nuclear radius. The uptake and the percentage of internalized activity of [^111^In]In-Pr were used to calculate the ^225^Ac disintegrations in each cell compartment (surface (CS), cytoplasm (Cy), nucleus (N)) at measured time points (1 h, 2 h, 6 h, and 24 h). It was assumed that the uptake and subcellular activity distribution of ^111^In were the same as those of ^225^Ac. The total absorbed dose to the nucleus of the SW1222 cells from ^225^Ac was calculated as a sum of the self-dose from activity in the cells, the cross-dose from the medium during the labeling period, and the cross-dose from the other cells during the colony-forming period. A detailed description can be found in Figure S2 and the supplementary data.

### Animal experiments

All animal experiments were done in accordance with protocols approved by the Institutional Animal Care and Use Committee of Memorial Sloan Kettering Cancer Center following National Institutes of Health guidelines for animal welfare.

To prepare the SW1222 CRC animal model used in biodistribution and therapy experiments, female athymic nude mice (strain: Hsd:Athymic Nude-Foxn^1nu^, Envigo, aged 6-8 weeks) were inoculated in the flank with SW1222 cells (5 x 10^6^ cells/mouse in 50% Matrigel (Corning)) 7-11 d prior to the experiments. The CLR37 PDX model originated from the biopsy of a liver metastasis from a patient with BRAF V600E CRC who had progressed through all standard treatment and was on trials of RAF + EGFR inhibitors. Notable findings from MSK IMPACT targeted exon sequencing included the BRAF V600E mutation + acquired NRAS G13R mutation 26. GPA33 expression in the original tumor and early-passage PDX was confirmed via GPA33-IHC. For CLR37 experiments, Balb/c Rag2^-/-^IL2Rγ^-/-^ mice (derived from the colony of Dr. Mamoru Ito, CIEA, Kawasaki, Japan, and now commercially available from Taconic as CIEA BRG mice) were used. Mice were randomly assigned to treatment groups.

### ^225^Ac-DOTA-PRIT regimen

^225^Ac-DOTA-PRIT was administered according to previously published protocol 6. In brief, 250 µg (1.19 nmol) of BsAb was administered 28 h before administration of [^225^Ac]Ac-Pr. Twenty-five μg (2.76 nmol) of CA were injected 24 h after BsAb injection and 4 h prior to [^225^Ac]Ac-Pr.

### Biodistribution and tissue dosimetry

To investigate the influence of administered [^225^Ac]Ac-Pr mass on *in vivo* PRIT performance, SW1222-xenografted mice were treated with anti-GPA33 BsAb, CA, and increasing amounts of [^225^Ac]Ac-Pr (7.8 kBq [0.21 µCi], 100-10000 pmol). A biodistribution study was performed 24 h post-injection (pi) of [^225^Ac]Ac-Pr (*n* = 4-5/mass dose). Mice were euthanized via CO_2_ asphyxiation, relevant tissues were collected, and activity content was measured at secular equilibrium.

For serial biodistribution studies, SW1222 xenograft-bearing mice were injected with anti-GPA33 or anti-HER2 BsAb and 37 kBq [1 µCi] of [^225^Ac]Ac-Pr (700 pmol) according to the protocol described above. Biodistribution was performed at 2 h, 24 h, 72 h, and 168 h pi of [^225^Ac]Ac-Pr (*n* = 5 mice/timepoint).

Time-integrated activity (TIA) was calculated by trapezoidal integration over the period from 0 to 168 h. Tissue activity concentration was assumed to be 0 Bq/mL at time 0 h. Time-activity curves were fit to exponential decay models, and tissue TIAs from 168 h to infinity were calculated using the exponential fits. The exception was the liver which had an increasing time-activity curve at 168 h. In this case, only physical decay was assumed to occur after 168 h. For all tissues, absorbed dose was calculated based on total local absorption of alpha and beta emissions only. Cross-dose from gamma emissions was excluded.

### *In vivo* therapy in a flank model of CRC

On 11 d post-inoculation of SW1222 xenografts, mice were randomized into groups of *n* = 10 and treated with anti-HER2 and anti-GPA33 ^225^Ac-DOTA-PRIT (37 kBq [1 µCi], 740 pmol) with appropriate controls. For GPA33, additional dose escalation therapy studies were conducted (all groups *n* = 10). These studies included a two-cycle treatment (total administered [^225^Ac]Ac-Pr: 74 kBq [2 μCi], cycle 1: 740 pmol, cycle 2: 790 pmol) separated by 1 week. Additionally, the efficacy of anti-GPA33 ^225^Ac-DOTA-PRIT (74 kBq [2 µCi], 700 pmol) was evaluated. Median survival (MS; in d after treatment initiation) was defined as time to tumor progression to a diameter of 15 mm or death for any reason. Tumor sizes were manually measured 1-2 times per week using a caliper, and tumor volumes were computed assuming ellipsoidal geometry.

Body weight and overall well-being of the mice were monitored to assess treatment-related toxicity. Mice were subjected to regular blood sampling, and samples were analyzed for white blood count (WBC), red blood cell count (RBC), and platelet count (PLT) using the Heska Element HT5 (Heska Corporation, Loveland, CO, USA).

Animals alive 124 d post treatment were evaluated for radiation-induced histological organ damage by board-certified veterinary pathologists (Laboratory for Comparative Pathology at Memorial Sloan Kettering Cancer Center and Weill Cornell Medical College, New York, NY, USA). If available, residual tumors of surviving mice were stained for expression of GPA33 and HER2. For details see supplemental material.

### Biodistribution and experimental therapy in a PDX model

*In vivo* biodistribution and therapy experiments were also performed with the CLR37 PDX model. For biodistribution, mice (*n* = 4) were injected with the anti-GPA33 ^225^Ac-DOTA-PRIT (74 kBq [2 μCi] of [^225^Ac]Ac-Pr) and sacrificed at 24 h pi. For the experimental therapy, mice (*n* = 5 mice/group) were treated with a single cycle of anti-GPA33 ^225^Ac-DOTA-PRIT (700 pmol, 37 kBq [1 µCi] or 74 kBq [2 μCi]). A [^225^Ac]Ac-Pr only (74 kBq [2 μCi], 700 pmol) and a non-treated control group were included. Survival criteria were the same as for the SW1222 flank model.

### Tumor and kidney autoradiography using iQID camera

Activity distribution in SW1222 xenografts and kidneys was visualized using an ionizing-radiation Quantum Imaging Detector (iQID) imaging system (QScint Imaging Solutions). For sample preparations, SW1222-tumor bearing mice were injected with anti-GPA33 or anti-HER2 ^225^Ac-DOTA-PRIT. Xenografts and kidneys were collected 2 and 24 h after [^225^Ac]Ac-Pr administration and immediately frozen in OCT. Frozen tissue sections (12 µm) were prepared using a cryostat microtome and placed on a ZnS:Ag phosphor Screen (EJ-440-100-3.2, Eljen Technology, Sweetwater, TX, USA). iQID digital autoradiographs (effective pixel size 19.5 microns) were generated by imaging tissue sections for 24 h in an alpha-particle counting mode. Counts images and ROI selections and statistics were processed using ImageJ.

### Statistical analysis

For biodistribution data, statistically significant differences (p < 0.05) were determined by one-way ANOVA with post-hoc t-test corrected for multiple comparisons with the Tukey method using GraphPad Prism 9 (GraphPad, Inc.), unless stated otherwise. Survival analysis was performed using GraphPad Prism 9. Kaplan-Meier survival curves were analyzed with the log-rank (Mantel-Cox) test. A p-value of < 0.05 was considered statistically significant.

## Results

### Multicellular dosimetry using MIRDcell and *in vitro* cell survival

Table 1 shows the summary of cellular dosimetry results for the clonogenic cell survival assay for each antibody type. For both GPA33 and HER2, the self-dose and cross-dose from the medium are about 72% and 28%, respectively. Overall, the estimated absorbed dose to cells treated with the anti-GPA33 ^225^Ac-DOTA-PRIT regimen was double that with the anti-HER2 regimen.

**Figure 1:**
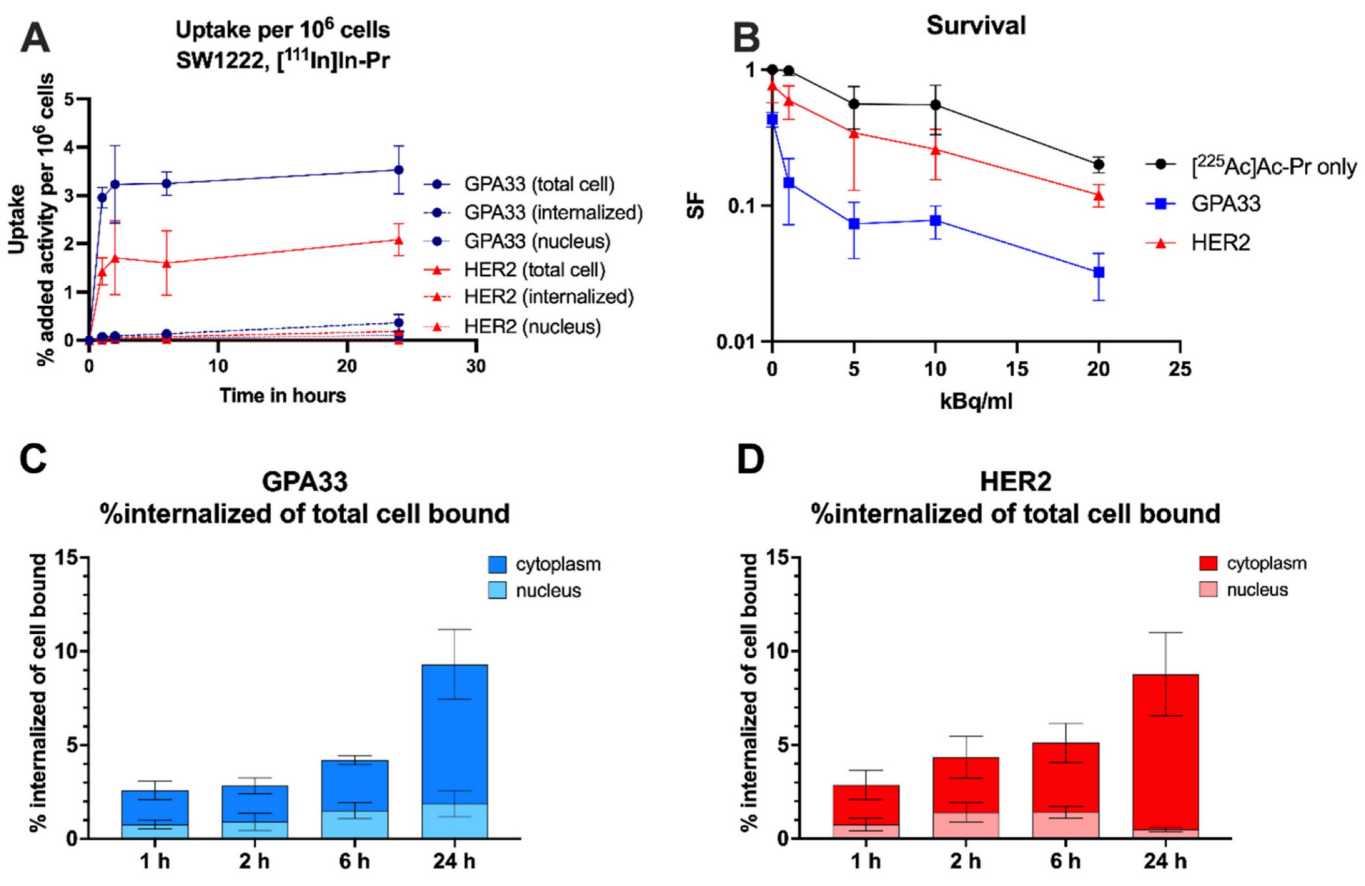
*In vitro* characterization of the anti-GPA33 and anti-HER2 DOTA-PRIT systems in SW1222 CRC cells. (A, C, D) Cells were incubated with 64 nM BsAb for 1 h at 37 ºC, followed by continuous incubation with 22 nM of [^111^In]In-Pr at 37 ºC (as a surrogate for ^225^Ac). (A) Uptake and internalization in adherent cells as % added activity (average ± SD). (C, D) activity internalized in adherent cells and normalized to total bound activity at each timepoint and separated into nucleus and cytoplasm. (B) Surviving fraction (SF) of SW1222 cells in suspension after incubation with [^225^Ac]Ac-Pr in the presence or absence of BsAb (average ± SD). Cells were incubated with 64 nM BsAb for 1 h on a rocker roller, followed by 90 min incubation with [^225^Ac]Ac-Pr before they were plated for colony formation. n = 3-6 per data point. Data from two independent experiments.

Exposure of SW1222 to anti-GPA33 and anti-HER2 ^225^Ac-DOTA-PRIT resulted in increased cell death (Figure 1). Greater potency was observed with the GPA33-targeting regimen. After incubation with 20 kBq/mL [^225^Ac]Ac-Pr the surviving fraction was 4-fold higher for cells that were pre-targeted with the HER2 BsAb. See Table S2 and Figure S3 for mean absorbed doses received by SW1222 cell nucleus during clonogenic survival assay.

### Slow internalization of [^111^In]In-Pr independent of the targeted antigen

Similar uptake and internalization patterns were observed for [^111^In]In-Pr in the presence of anti-GPA33 and anti-HER2 BsAb in SW1222 cells with stable total uptake of [^111^In]In-Pr after 2 h but increasing internalization with time (Figure 1, Table S3). There were no significant differences in the internalized fraction or nucleus-associated radioactivity between the two targets at any of the investigated time points. At 24 h, the internalized fractions were 9 ± 1% and 9 ± 2% of the total cell-bound activity for GPA33 and HER2, respectively. Absolute uptake was 1.86-fold higher (p < 0.05) when targeting GPA33, attributed to its higher expression level in SW1222 cells.

In LS174T cells (Figure S4, Table S3), the total cell uptake continued to increase until the 24 h timepoints for both GPA33 and HER2. While there was no significant difference in internalization between cell lines for HER2, the internalization of [^111^In]In-Pr and its localization in the nucleus were significantly higher for GPA33 BsAb at all time points, except at 6 h.

Refer to Table S3 for a detailed analysis of uptake and internalization results, including statistical comparisons for the SW1222 and LS174T cell lines.

### *In vivo* targeting and dosimetry

**Figure 2:**
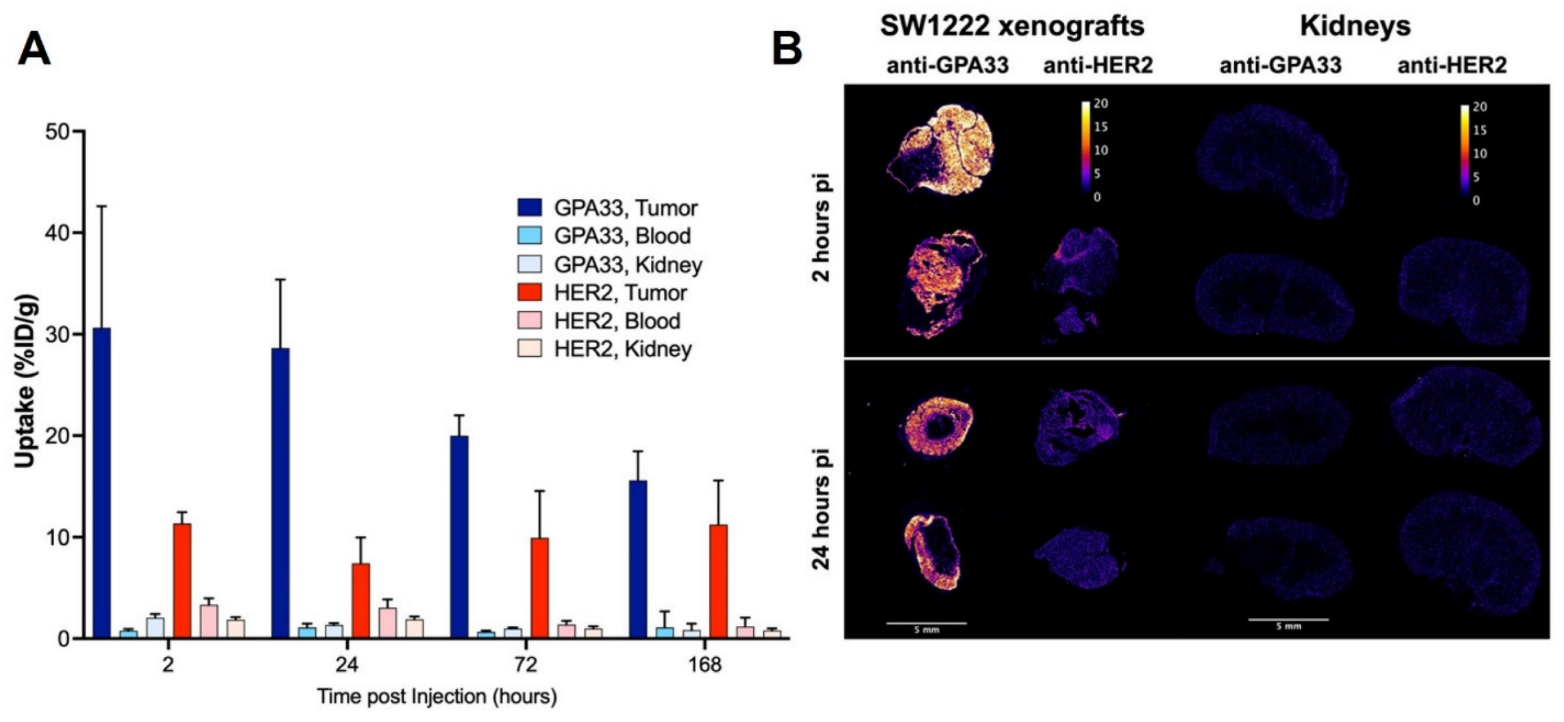
(A) Serial biodistribution of [^225^Ac]Ac-Pr (700 pmol) in SW1222 xenografted mice (n = 4-5 mice/time point, average ± SD). Uptake over time in tumors, blood, and kidney after pre-targeting with anti-GPA33 or anti-HER2 BsAb. Full biodistribution data can be found in Figure S6 and Tables S6 and S7. (B) Tumor and kidney autoradiography using iQID camera 2 h and 24 h after injection of [^225^Ac]Ac-Pr. Tissues from two mice per timepoint, and target, except for anti-HER2 2 h (only 1 mouse). Scalebar: 5 mm. Colorbar is in units of alpha counts acquired during 24 h imaging acquisition.

The [^225^Ac]Ac-Pr dosing experiment targeting GPA33 showed that the highest tumor-to-kidney ratios could be achieved at [^225^Ac]Ac-Pr mass doses between 350 and 700 pmol (Figure S5, Tables S4 and S5). A radiohapten dose of 700 pmol was used for serial biodistribution in mice bearing SW1222 xenografts using either the anti-GPA33 or anti-HER2 ^225^Ac-DOTA-PRIT regimen (Figure 2, Figure S6, Tables S6 and S7). Peak tumor uptake was achieved rapidly, as a maximum average uptake of 31 ± 12 %ID/g or 11 ± 1 %ID/g was observed at 2 h pi for targeting GPA33 and HER2, respectively. Tumor retention of [^225^Ac]Ac-Pr was higher when targeting HER2 with no significant release of activity over the investigation period. Uptake of [^225^Ac]Ac-Pr in mice pre-targeted with anti-HER2 BsAb was significantly higher in most tissue at 2 h, 24 h, and 72 h. Most notably, the concentration of ^225^Ac in blood was 2- to 4-fold higher in HER2-targeted mice than in GPA33 between 2 h and 72 h pi. Kidney uptake of [^225^Ac]Ac-Pr for the anti-HER2 ^225^Ac-DOTA-PRIT was twice the uptake of mice that received the anti-GPA33 ^225^Ac-DOTA-PRIT regimen. See Tables S6 and S7 for all statistical comparisons. Autoradiography of SW1222 xenografts revealed an apparent uniform distribution of ^225^Ac for both target antigens (Figure 2). Ratios of tumor to kidney activity (Table 2) were obtained via analysis of iQID alpha counts digital autoradiography images (Table S8). Based on the iQID kidney images collected at 2 and 24 h pi, the ^225^Ac uptake is concentrated in the cortex. In general, tumor-to-kidney ratios obtained via *ex vivo* biodistribution tended to be higher.

The *ex vivo* serial biodistribution data was used to estimate the absorbed doses to tumors and healthy tissues (Table 3, Table S9). The estimated dose to the SW1222 xenograft was about 1.7-fold higher when targeting GPA33 compared with HER2. Not considering the tumors, the absorbed doses were highest for blood, kidney, and liver, with doses being generally lower with the GPA33 targeting regimen than the HER2 regimen.

### ^225^Ac-DOTA-PRIT results in significantly prolonged survival in a flank model of human CRC with histological cures

**Figure 3:**
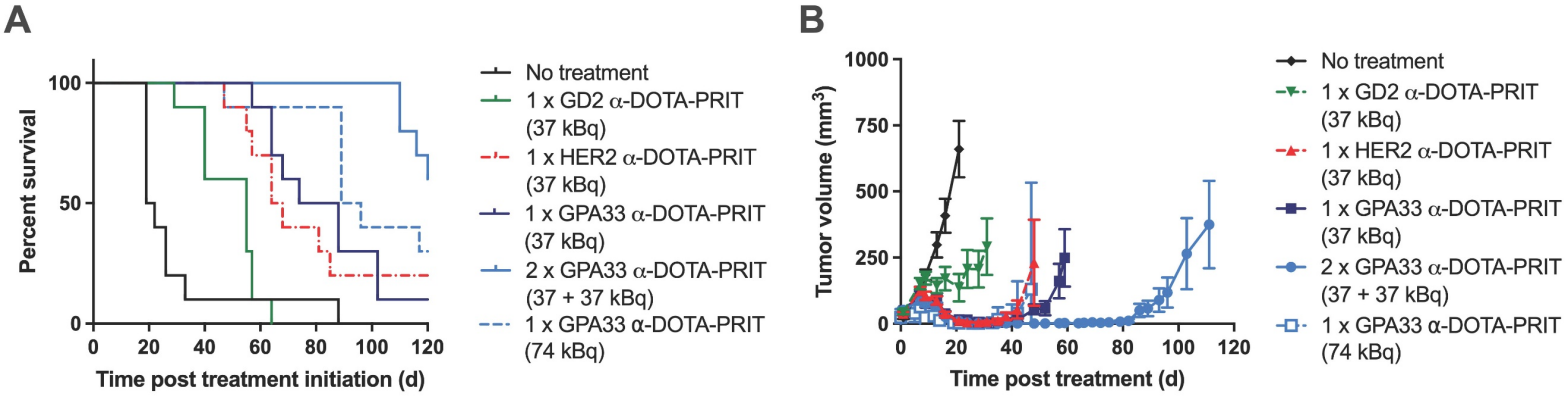
Experimental therapy results from mice bearing SW1222 xenografts after different ^225^Ac-DOTA-PRIT treatment regimens (n = 10/group). (A) Percent survival (B) Tumor volumes (mm^3^). Graphs were discontinued after exclusion of the first animal in each group. Please see Table 4 for detailed information on long-term survivors and tumor status.

Survival curves, tumor growth curves, median survival, and residual tumor volumes of animals alive 124 d post-treatment are shown in Figure 3 and Table 4. All groups treated with the ^225^Ac-DOTA-PRIT regimen, including the off-target control, had significantly prolonged survival compared with the no treatment group. All treatments were well tolerated, with only a transient average body weight loss of less than 10% (Figure S7). WBC, RBC, and PLT counts remained within the reference range for nude mice during the first month after treatment, indicating the absence of acute toxicity (Figure S8).

For single-cycle treatments (37 kBq) of either anti-GPA33 ^225^Ac-DOTA-PRIT or anti-HER2 ^225^Ac-DOTA-PRIT, all treated mice showed complete response (CR; tumor volume ≤10 mm^3^) at approximately 20 d post-treatment; however, tumor escape was observed for most of the animals. For anti-GPA33 therapy, a single mouse was still alive at 124 d with a tumor volume of 96.2 mm^3^. For anti-HER2 therapy, 2/10 were still alive with tumor volumes of 0 mm^3^ (*i.e.*, no palpable tumor), and 141.7 mm^3^. There was no significant difference in MS between the single-cycle anti-HER2 and anti-GPA33 treatments (all individual p-values in Table S10). Two cycles of anti-GPA33 ^225^Ac-DOTA-PRIT significantly prolonged MS compared with all other groups, except the high single dose (74 kBq) of anti-GPA33 ^225^Ac-DOTA-PRIT (all individual p-values in Table S10). Similar to single-cycle treatment (37 kBq), all treated mice (10/10) showed CR, and 5/10 mice of this group were still alive after 124 d, two of which had histological cures. Histological cures at 124 d post treatment were also observed in the 1 x 74 kBq anti-GPA33 (3/5 mice alive at endpoint) and 1 x 37 kBq anti-HER2 (1/2 mice alive at endpoint) ^225^Ac-DOTA-PRIT groups. Examples of hematoxylin and eosin-stained, GPA33-stained, and HER2-stained residual tumors are shown in Figure S9.

Kidney damage was scored according to criteria in Jaggi *et al.* 27. Results can be found in Table S11. No major pathological changes were observed in the surviving mice, and there was no difference between groups. The minimal tubulointerstitial changes were interpreted as incidental changes that cannot be differentiated from spontaneous background lesions commonly seen in murine kidneys. These findings were not considered adverse as they did not appear to significantly affect renal function or general health of the animals, based on the low percentage of the renal parenchyma affected (<1%), normal serum chemistry parameters, and absence of clinical signs or alterations in body weights. A representative kidney image can be found in Figure S10.

### Anti-GPA33 ^225^Ac-DOTA-PRIT shows efficacy in a GPA33 positive patient derived xenograft model

Expression of GPA33 in the CLR37 PDX was confirmed by IHC (Figure S11). Biodistribution of [^225^Ac]Ac-Pr 24 h pi (Figure S12) showed efficient targeting of GPA33 in CLR37 PDX, with the tumor uptake being 16 ± 7 %IA/g 24 h pi.

**Figure 4:**
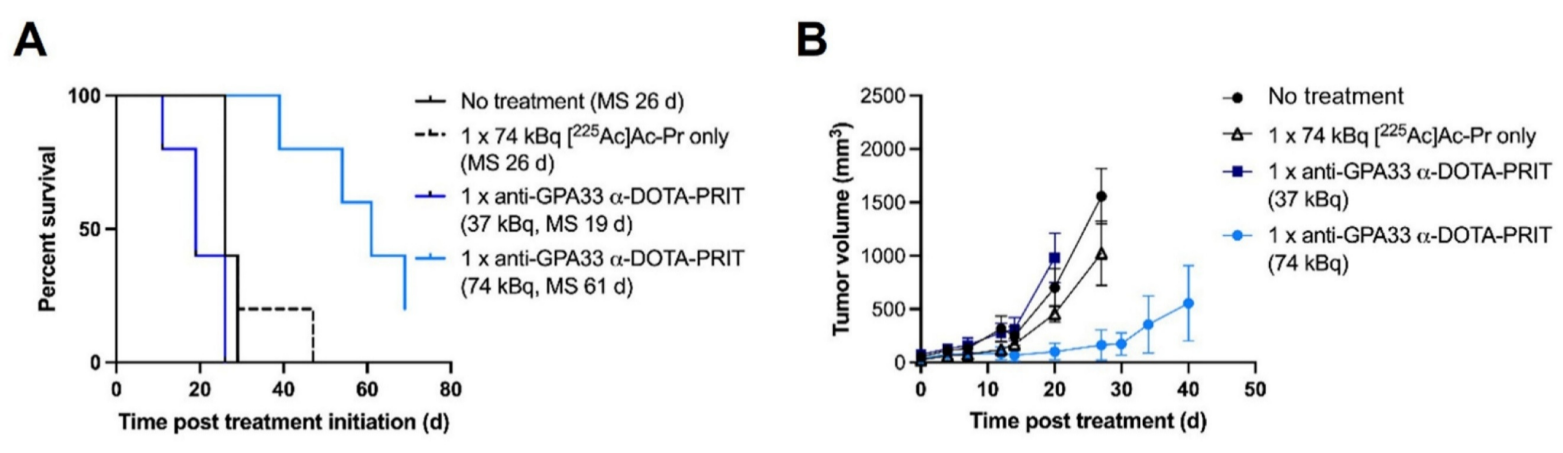
Therapy of mice bearing GPA33 expressing PDX. (A) Survival curves after experimental anti-GPA33 ^225^Ac-DOTA-PRIT of CLR37 PDX in mice (n = 5 mice/group). (B) Tumor volumes (mm^3^). Graphs were discontinued upon exclusion of the first animal.

Treatment of CLR37 PDX with 74 kBq anti-GPA33 ^225^Ac-DOTA-PRIT significantly delayed growth of CLR37 PDX and significantly prolonged survival compared with all other treatment groups (Figure 4). There was no significant difference in MS between no treatment, [^225^Ac]Ac-Pr only, and 37 kBq anti-GPA33 ^225^Ac-DOTA-PRIT groups. No changes in body weight were observed (Figure S12).

## Discussion

Herein we evaluated ^225^Ac-DOTA-PRIT against GPA33, an antigen highly expressed in CRC, and HER2, an antigen with only 20% of the comparable antigen concentration, in terms of treatment efficacy. Despite significant differences in tumor uptake, we found that a single cycle of pretargeting with 37 kBq of [^225^Ac]Ac-Pr achieved similar tumor control efficacy. Treatment of subcutaneous CRC SW1222 xenografts in mice with either anti-GPA33 or anti-HER2 ^225^Ac-DOTA-PRIT resulted in CRs and histological cures in a subset of the long-term survivors without apparent toxicity to healthy organs. Due to the excellent TIs, more aggressive treatment regimens (either 1 x 74 kBq or 2 x 37 kBq) further enhanced therapeutic efficacy and prolonged survival without detectable toxicity. The MTD has not yet been reached. We also verified the efficacy of anti-GPA33 ^225^Ac-DOTA-PRIT in a CRC PDX. This model represents heavily pre-treated CRC and was generated from a patient with resistant disease to both chemotherapy and targeted therapy. In summary, these studies establish anti-GPA33 and anti-HER2 ^225^Ac-DOTA-PRIT as safe and effective treatments for CRC co-expressing GPA33 and HER2.

The potency of the anti-HER2 regimen in comparison with the anti-GPA33 regimen was somewhat surprising. This phenomenon has been documented previously by Boudousq *et al.* during RIT treatment of small volume carcinomatosis using two different ^212^Pb-IgG; the ^212^Pb-35A7 mAbs (anti-carcinoembryonic antigen) were less efficient than ^212^Pb-trastuzumab (anti-HER2), although the dose absorbed by the tumor was higher for ^212^Pb-35A7 28. Several potential mechanisms may be at play in this setting. One possibility is that the relative biological effectiveness (RBE) differs due to the intrinsic biology of the two antibody-antigen systems, perhaps leading to increased bystander effects 29-32. Another possibility is that this reflects the intrinsic heterogeneity of each tumor-associated antigen on individual tumor cells 33. Nonetheless, for a given cGy delivered to the nucleus, HER2 appears to be more effective than GPA33 as a carrier for delivering cytocidal α-radiation.

The pathophysiology of the SW1222 solid tumor model provides a unique opportunity to evaluate the radiation dose-response relationships of these two antigen targets. This model is highly vascularized and well-differentiated 34. We investigated the intratumoral distribution of ^225^Ac using autoradiography with an iQID alpha camera 35, 36. Images indicated relatively homogenous uptake independent of the targeted antigen. This targeting pattern allows for a fair comparison of the two antigens, especially in the context of TAT. Therefore, differences in the effectiveness of HER2 and GPA33 could not be attributed to obvious heterogeneity of uptake within the tumor mass.

To understand the radiation biology underlying the enhanced effectiveness of anti-HER2 ^225^Ac-DOTA-PRIT compared to anti-GPA33 ^225^Ac-DOTA-PRIT, we conducted *in vitro* studies and estimated the nuclear doses using MIRDcell techniques. We observed a two-fold difference in doses: 1.3 Gy for GPA33 and 0.65 Gy for HER2 for the same activity concentration in the medium. Notably, both regimens showed similar *in vivo* efficacy, despite this apparent difference in nuclear dose and the saturating *in vitro* cell survival curves. This observation supports a testable hypothesis: a threshold radiation dose to the nucleus may be required to achieve a CR. To further investigate this phenomenon, we intend to repeat these studies with larger cohorts and a broader range of administered activities, with the specific objective of determining whether this effect is maintained at lower ^225^Ac doses or when using a low linear energy transfer radioisotope with a broader dynamic dosing range. We hypothesize that, despite likely differences in antibody-antigen complex trafficking within the cell due to GPA33 or HER2 antigen, tumor cell death can be expected if the radiation dose delivered to the nucleus exceeds a critical threshold. A corollary of this idea is that the delivery of the increased amount of radiation absorbed dose from α-particles beyond this threshold is in fact redundant and probably toxic.

While α-dosimetry and the prediction of radiobiological effects are complex and continuously discussed within the TAT community 37, 38, we attempted to establish dose-response relationships for the two regimens. In terms of the average number of sites per cell, there is a difference on the order of 5-fold between the two antigens. Our macrodosimetry estimation predicted a 1.7-higher dose to be delivered to GPA33-targeted tumors (193.31 Gy/37 kBq; RBE = 5) compared to HER2-targeted tumors (113.91 Gy/37 kBq; RBE = 5). However, at equal administered [^225^Ac]Ac-Pr, the therapeutic outcomes were similar. This suggests that relative efficacy could not be predicted by absolute radiation absorbed dose (Gy).

A key factor that could explain the enhanced potency of HER2 compared to GPA33 likely relates to differences at the cellular level and the contrasting intrinsic biology of the two tumor-associated antigens. GPA33 is associated with tight junctions between cells and is minimally endocytosing 14, 15. In contrast, HER2 is a part of the cell's growth and proliferation signal transduction cascade and is known to be an endocytosing antigen 39. Additionally, since the anti-HER2 BsAb contains the sequence for trastuzumab, it may have a similar signal-blocking function, providing an additional anti-tumor effect 40. This was previously observed in high HER2-expressing BT474 human breast cancer xenografts, although the standalone effect of the anti-HER2 BsAb was small (MS: 62 d for no treatment control, 72 d for BsAb only, and >130 d for anti-HER2 ^225^Ac-DOTA-PRIT) 6. These biological differences likely have a significant impact on the RBE.

Efficacy and therapeutic index (dose-toxicity relationships) are equally important. The data presented here is another step towards establishing dose-toxicity benchmarks for TAT and continues to push preclinical boundaries. Despite careful histologic examination of the kidneys, nephrotoxicity was not observed, and thus further dose escalation is feasible. Our dosimetry predictions, based on serial biodistribution data, estimated the dose to kidneys to be 10.5 Gy/37 kBq (RBE = 5). We acknowledge that microdosimetry of key organs at risk, including the kidneys, is very important, and we will continue to characterize nephrotoxicity with great care in future studies.

The findings and paradigms established in this study open the door to several possible future directions. While GPA33 and HER2 remain compelling CRC targets, our murine xenograft models do not fully capture potential human toxicity profiles. Further preclinical and early clinical trials in man are needed to evaluate on-target, off-tumor effects—such as those that may arise from GPA33 expression in normal gut tissue. In humans, radiation absorbed dose delivered by the GPA33-directed radioimmunoconjugate _131_I-huA33 was readily tolerated in the range of 150 cGy or less to the red marrow in most patients 41. We will keep these threshold numbers in mind as we plan for early clinical trials in man with GPA33-DOTA-PRIT. Furthermore, co-targeting these or additional co-expressed tumor antigens may help overcome the issue of antigen and uptake heterogeneity. The versatility of our DOTA-PRIT makes it an excellent candidate for co-targeting multiple antigens using an antibody cocktail, with the same radioligand binding to each BsAb. Optimized antibody cocktails can substantially enhance cell-absorbed doses compared to conventional single-target TAT 33. Additionally, our recently developed self-assembling and disassembling (SADA) BsAb platform 42 offers further potential to improve TIs and eliminate the need for a CA.

## Conclusion

In conclusion, we have demonstrated comparable safety and effectiveness for ^225^Ac-DOTA-PRIT against two different antigen systems, GPA33 and HER2. These treatment regimens resulted in significant prolonged survival and histologic cures in a subset of human CRC models growing as xenografts in immunodeficient mice. These outcomes were achieved with well- tolerated doses of ^225^Ac-DOTA-PRIT. These findings point to the potential of effective TAT regimens in advanced CRC using one or more tumor-associated antigen targets, either sequentially or in combination.

## Supplementary Material

Supplementary materials and methods, figures and tables.

## Figures and Tables

**Table 1:** Summary of absorbed doses to cell nucleus of SW1222 cells based on uptake and internalization data and calculated using MIRDcell V4.

Source of Dose and Stage of Colony Forming Assay	*D* (Gy per kBq/mL)	
	GPA33	HER2
Self-dose during labeling	0.04	0.02
Cross-dose from medium during labeling	0.024	0.024
Self-dose during colony forming	0.90	0.45
Cross-dose during colony forming	0.371	0.185
Total absorbed dose (self + cross)	**1.3**	**0.65**

**Table 2:** Quantification of iQID autoradiography images. Ratios are based on ROI intensities. Data in column 1-3 represents one animal per row. Absolute ROI intensities can be found in Table S8.

Target and timepoint	Mean IntensityTumor/Cortex Ratio	Mean Intensity Tumor/Medulla Ratio	Mean Intensity Tumor/Kidney Ratio
GPA33 2 h pi	19.1	68.4	14.9
	7.6	43.7	6.5
GPA33 24 h pi	7.5	68.0	6.7
	7.1	32.7	5.8
HER2 2 h pi	1.6	2.4	1.0
HER2 24 h pi	1.8	3.8	1.2
	1.5	4.6	1.1

**Table 3:** Mean absorbed doses delivered to tissues and therapeutic indices based on serial biodistribution of ^225^Ac-anti-GPA33 and ^225^Ac-anti-HER2-DOTA-PRIT in SW1222 xenografted mice. Dose shown as Gy/37 kBq. Estimation does not include cross-dose between organs, total local absorption assumed. RBE: relative biological effectiveness

Tissue	Tissue doses (Gy/37 kBq)					
	GPA33		HER2		TI	
	^225^Ac + all daughters	^225^Ac + all daughters (RBE = 5)	^225^Ac + all daughters	^225^Ac + all daughters (RBE = 5)	GPA33	HER2
Blood	2.18	10.89	3.16	15.78	17.7	7.2
**Tumor**	**38.66**	**193.31**	**22.78**	**113.91**		
Heart	0.64	3.19	1.14	5.70	60.5	20.0
Lungs	1.08	5.38	1.70	8.50	35.9	13.4
Liver	1.70	8.50	2.77	13.85	22.8	8.2
Spleen	0.88	4.40	1.65	8.24	44.0	13.8
Stomach	0.17	0.85	0.29	1.45	228.1	78.5
Sm. Intestine	0.26	1.31	0.46	2.28	148.1	49.9
Lg. Intestine	0.35	1.73	0.41	2.05	111.6	55.6
Kidneys	2.02	10.10	2.10	10.50	19.1	10.9
Muscle	0.34	1.69	0.51	2.54	114.7	44.9
Bone	0.73	3.63	0.76	3.81	53.3	29.9

**Table 4:** Summary of experimental therapy results from SW1222-xenografted mice: MS, residual tumor volume, and tumor status from mice surviving until 124 d post-treatment. Histological cures were determined at study endpoint based on hematoxylin and eosin staining and assessment by a board-certified veterinary pathologist.

Treatment Group	MS (d)	Alive at 124 d (residual tumor volume)	Tumor Status
No treatment	20.5^a^	0/10	n/a
1 x ^225^Ac-anti-**GD2**-DOTA-PRIT (37 kBq)	55^a,b^	0/10	n/a
1 x ^225^Ac-anti-**HER2**-DOTA-PRIT (37 kBq)	66^b^	2/10 (0 mm^3^, 141.7 mm^3^)	1: Histologic “cure” 2: Carcinoma, with necrosis
1 x ^225^Ac-anti-**GPA33**-DOTA-PRIT (37 kBq)	81^b^	1/10 (96.2 mm^3^)	1: Carcinoma, with necrosis
2 x ^225^Ac-anti-**GPA33**-DOTA-PRIT (37 + 37 kBq)	123^a,b^	5/10 (0 mm^3^, 0 mm^3^, 11.5 mm^3^, 62.8 mm^3^, 172.5 mm^3^)	1 and 2: Histologic “cure” 3: Carcinoma, with lymphocytic infiltrates 4: Carcinoma, with necrosis 5: Carcinoma, with necrosis
1 x ^225^Ac-anti-**GPA33**-DOTA-PRIT (74 kBq)	92.5^b^	3/10 (0 mm^3^, 0 mm^3^, 19.2 mm^3^)	1,2,3: Histologic “cure” 3: Mass of with intratumoral necrosis, mucinous material, and mineralized concretions, surrounded by chronic inflammation and fibrosis (no neoplastic cells identified)

^a^ significant difference p < 0.05 compared with 1 x ^225^Ac-anti-GPA33-DOTA-PRIT (37 kBq) by Log-rank (Mantel-Cox) test; ^b^ significantly prolonged survival compared to no treatment group by Log-rank (Mantel-Cox) test.
