# Supplementary material for: Comparison of targeting two antigens (GPA33 versus HER2) for 225Ac-pretargeted alpha-radioimmunotherapy of colorectal cancer
Source: Theranostics. 2025 Jun 20;15(15):7489–500. doi: 10.7150/thno.116062 (PMC12315807; doi:10.7150/thno.116062)
Supplement: Supplementary file 1 — Supplementary materials and methods, figures and tables. [file thnov15p7489s1.pdf]

# Supplementary Data

## Comparison of targeting two antigens (GPA33 versus HER2) for $^{225}$Ac-pretargeted alpha-radioimmunotherapy of colorectal cancer

*Binding affinities of BsAb*

Table S1: Binding affinities of BsAb to target antigens measured by surface plasmon resonance (Biacore T100).

| BsAb | Antigen | ka (1/Ms) | kd (1/s) | KD (M) |
|---|---|---|---|---|
| anti-GPA33-C825 | GPA33 | 9.15E+05 | 5.81E-03 | 6.35E-08 |
| | C825 | 1.90E+04 | 2.20E-04 | 1.16E-08 |
| anti-HER2-C825 | HER2 | 8.95E+05 | 7.85E-06 | 8.77E-12 |
| | C825 | 2.10E+04 | 1.26E-04 | 6.00E-09 |

*Cellular Uptake and Internalization of [$^{111}$In]In-Pr*

SW1222 cells were plated into 6-well plates at a density of $10^6$ cells/well one day before the experiment. One extra plate was prepared, designated for cell counting at the end of the experiment. On the day of experiment cells were first incubated with 64 nM of BsAb (anti-HER2 or anti-GPA33, in a total volume of 0.5 mL/well). After 1 hour at 37 °C the antibody solution was removed, cells were washed once with 1 mL of PBS. Thereafter 22 nM of [$^{111}$In]In-Pr (in a volume of 0.5 mL) was added. Cells were incubated at 37 °C until pre-determined time points. At each timepoint a set of 3 wells/group was removed from the incubator. The [$^{111}$In]In-Pr solution was removed and cells were washed two times with1 mL of PBS. Subsequently, 0.5 mL of stripping buffer (0.2 M glycine, 0.15 NaCl, 4 M urea, pH 2) was added for 5 min on ice. After incubation the stripping buffer was collected, and its activity content was considered membrane bound. The activity remaining with the cells was considered internalized. The $^{111}$In-activity content in the membrane and internalized fractions were measured using an automated gamma counter (Wallac Wizard 1470, Wallac Oy, Turku, Finland)

*Cellular Dosimetry for Colony Forming Assay*

**Colony forming assay**

For the cell survival studies, SW1222 cells were incubated with antibody and radioactivity in culture tubes on a rocker roller at 37 °C (Figure S1). During both incubation steps the cell concentration was $0.5 \times 10^6$ cells/mL in a volume of 1 mL. The cells were first incubated with the antibody, washed with PBS, centrifuged, and resuspended to a concentration of $10^6$ cells/mL. Then 0.5 mL of antibody-labeled cells ($0.5 \times 10^6$ cells) were transferred into fresh culture tubes. A volume of 0.5 mL of media containing the radioligand ([$^{225}$Ac]Ac-Pr) was then added over a range of activity concentrations. The total incubation volume of 1 mL was maintained for all final activity concentrations (0, 1, 5, 10, 20 kBq/mL). The tubes were then placed on a rocker-roller and incubated for 90 min. Afterwards, the cells were washed and plated for colony formation in 6 well plates.

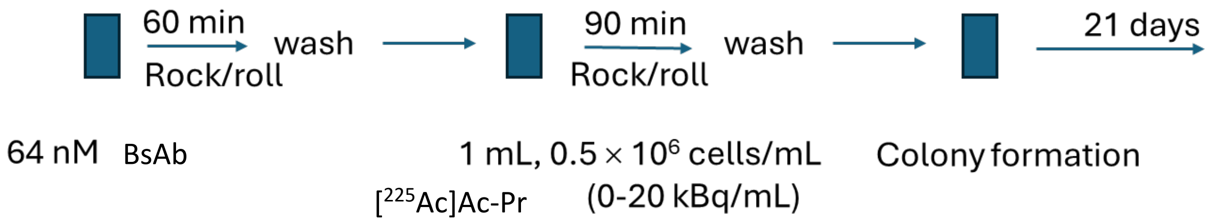


**Figure S1:** Cell survival assay. Experimental timeline for SW1222 cells treated with [$^{225}$Ac]Ac-Pr and subsequently seeded for colony formation.

## Cellular dosimetry

*Parameters and assumptions*

1) *Cell dimensions*. Radii of cell and cell nucleus for SW1222 cells: RC = 6 µm, RN = 4 µm.
2) *Cell concentration*. Treatments were conducted in 1 mL at a concentration of $0.5 \times 10^6$ cells/mL.
3) *Cellular uptake*. Based on Figure 1A in the manuscript, approximately 3% and 1.5% of activity in the medium is bound to cells after 90 min for GPA33 and HER2, respectively.
    1) $A_0$ = Activity/cell in Bq/cell per kBq/mL = $C$ (kBq/mL) × 1000 Bq/kBq × %uptake / cell concentration cells/mL).
    2) $A_0$(GPA33) = $C$ (kBq/mL) × 1000 Bq/kBq × 0.03 / $0.5 \times 10^6$ cells/mL = $0.6 \times 10^{-4}$ Bq/cell per kBq/mL.
    3) $A_0$(HER2) = $0.3 \times 10^{-4}$ Bq/cell per kBq/mL.
4) *Internalization of activity during labeling.* Based on Figure 1C and 1D, internalization is less than 2% after 90 min in medium with [$^{225}$Ac]Ac-Pr and most of the internalized activity is in the cytoplasm. The self-dose $S(N<CS)$ and $S(N<Cy)$ are 0.12 Gy/Bq s and $S(N<Cy)$ = 0.184 Gy/Bq s, respectively. Therefore, internalization will not have a substantial effect on absorbed dose calculations during the 90 min labeling period. Hence, internalization is ignored for dosimetry calculations for the 90-min [$^{225}$Ac]Ac-Pr labeling period.
5) *Self-dose during labeling*. Binding of [$^{225}$Ac]Ac-Pr is complete in less than 90 min as per table above. Assume rate of binding of [$^{225}$Ac]Ac-Pr is instantaneous. Therefore, $D_{self}$(labeling) = 1.5 h × $A_0(t = 1.5\text{ h})$ $S(N<CS)$.
6) *Internalization of activity during colony forming*. Internalization was measured with [$^{111}$In]In-Pr over a period of 24 h. A rise to 2% occurs within 1 hour, and then increases linearly out to 9% at 24 h.
7) *Effective half-life of cellular activity during colony forming period*. During the colony forming period, the cellular activity will decrease with an effective half-time that corresponds to the combination of physical decay and biological clearance. Assume that biological clearance is dictated by the doubling time of the SW1222 cells which is 60 h. Therefore, $T_e$ = effective half-time = (60 h × 238 h)/(60 h + 238 h) = 48 h.
8) *Self-dose during colony forming period*. Given the long effective half-time, the distribution between cell surface, cytoplasm, and nucleus measured at 24 h will provide a reasonable estimate for absorbed dose calculations. $D_{self}$(cells in colony)= 1.44 $A_{cell}(t = 1.5\text{ h}) \times T_e \times S(N<N(2.9\%),Cy(7.4\%),CS(89.7\%))$. Ac-225 daughters are included for cytoplasm and nucleus localization. From MIRDcell V4.14, the $S$ coefficients are:
    a) $S(N<CS$, no daughters$)$=0.0342 Gy/Bq s

b) *S*(N<Cy, with daughters)=0.184 Gy/Bq s
c) *S*(N<N, with daughters)=0.532 Gy/Bq s

At $t$ = 24 h, fraction in cytoplasm is 0.074, fraction in nucleus is 0.029, fraction on surface is 0.897. Weighted *S* value = (0.0343×0.897) +(0.184 × 0.074) + (0.029 × 0.532) = 0.060 Gy/Bq s.

Using the parameters and assumptions cited above, the mean absorbed dose to the cell nucleus of the treated cells is calculated at each stage of the experiment as follows:

*Self-dose during labeling*

$$D_{self,labeling}(\text{GPA33}) = T_{\text{labeling}}\, \bar{A}_{\text{cell}}\left(t = T_{\text{labeling}}\right) S(\text{N} \leftarrow \text{CS}) =$$

$$1.5\text{ h } \times 3600\frac{\text{s}}{\text{h}} \times 0.6 \times 10^{-4}\text{ Bq per kBq/mL } \times 0.12\frac{\text{Gy}}{\text{Bq s}} = 0.04\text{ Gy per kBq/mL}$$

$$D_{self,labeling}(\text{HER2}) = 0.02\text{ Gy per kBq/mL}$$

*Cross-dose from culture medium during labeling*

Given that the dosimetry is dominated by alpha particles and their range in water corresponds to several cell diameters, assume that the mean absorbed dose to the cell nucleus is approximately equal to the mean absorbed dose to the culture medium.

Medium Volume = 1 mL (corresponds to radius = 0.62 cm if sphere of water). From MIRDcell V4.14, a 0.62 cm radius sphere with $^{225}$Ac + daughters gives the following *S* coefficient: *S*($^{225}$Ac+daughters) = 4.57× $10^{-9}$ Gy/Bq s.

$$D_{cross,labeling}(\text{cell} \leftarrow \text{medium}) = T_{\text{labeling}}\, \bar{A}_{\text{medium}}\, S(\text{cell} \leftarrow \text{medium}, {}^{225}\text{Ac} + \text{daughters}) =$$

$$1.5\text{ h } \times 3600\frac{\text{s}}{\text{h}} \times 1{,}000\text{ Bq} \times 4.57 \times 10^{-9}\frac{\text{Gy}}{\text{Bq s}} = 0.024\text{ Gy per kBq/mL}$$

*Self-dose during colony forming period*

$$D_{self,colony}(\text{GPA33}) = 1.44 \times 48\text{ h } \times 3600\frac{\text{s}}{\text{h}} \times 0.6 \times 10^{-4}\text{ Bq per kBq/mL } \times 0.060\frac{\text{Gy}}{\text{Bq s}} = 0.9\text{ Gy per kBq/mL}$$

$$D_{self,colony}(\text{HER2}) = 0.45\text{ Gy per kBq/mL}$$

*Cross-dose during colony forming period*

The cross-dose received by cells from other cells in the colony was estimated using the Multicellular Geometry < 2-D Colony tab in MIRDcell V4.14. A circular shape was selected for the colony with a radius of 16 μm which created a colony of 5 cells growing on a plane. The Source was set to Ac-225. The distribution of activity within the cell was set to the same as for the self-dose calculation. The Max mean Activity per cell was set to 0.6 × $10^{-4}$ Bq, and Time integrated activity coefficient was set to 69 h, which corresponds to complete decay with an effective half-time of 48 h. The cross-dose per kBq/mL was taken from the Output tab of MIRDcell V4.14.

$$D_{cross,colony}(\text{GPA33}) = 0.371\text{ Gy per kBq/mL}$$

$$D_{cross,colony}(\text{HER2}) = 0.185\text{ Gy per kBq/mL}$$

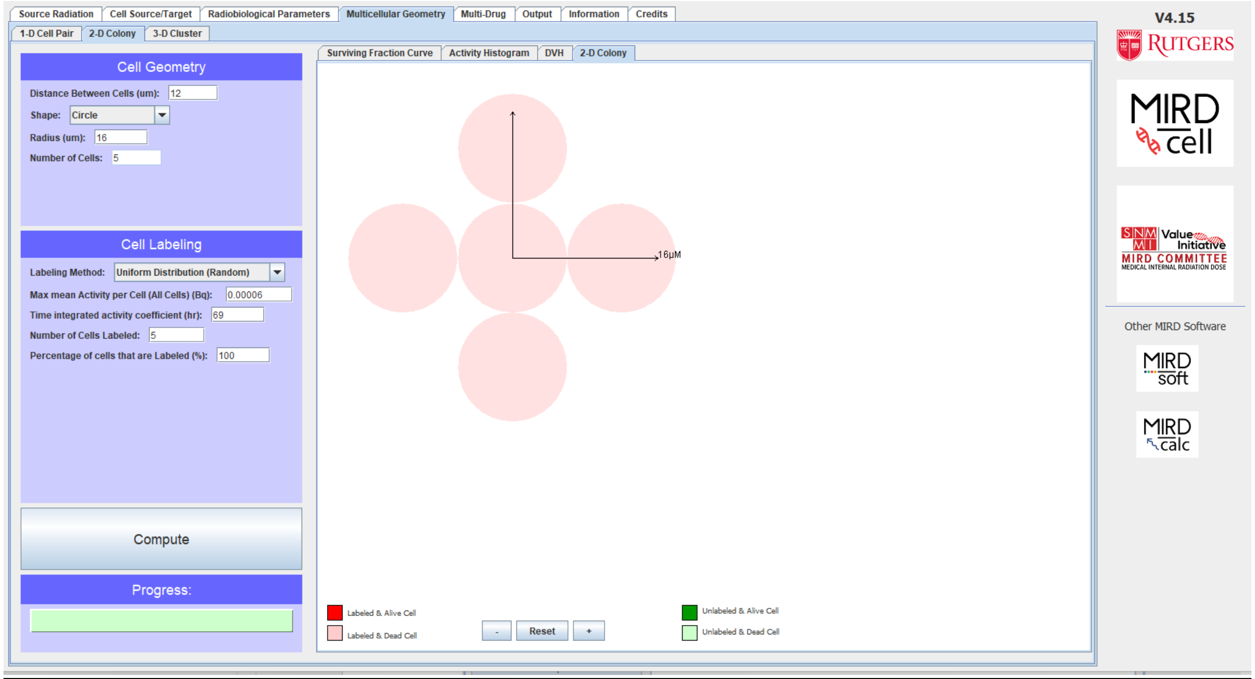


**Figure S2:** MIRDcell V4.14 setup for calculating cross absorbed dose to cells during the colony forming period.

*Total absorbed dose*

$$D_{total}(\text{GPA33}) = D_{self,labeling} + D_{cross,labeling} + D_{self,colony} + D_{cross,colony}$$
$$= 0.04 + 0.024 + 0.9 + 0.371 = 1.3 \text{ Gy per kBq/mL}$$
$$D_{total}(\text{HER2}) = D_{self,labeling} + D_{cross,labeling} + D_{self,colony} + D_{cross,colony}$$
$$= 0.02 + 0.024 + 0.45 + 0.185 = 0.65 \text{ Gy per kBq/mL}$$

**Table S2:** Mean absorbed doses received by SW1222 cell nucleus during clonogenic survival assay.

| Activity concentration (kBq/mL) | Absorbed Dose to GPA33 Cell Nucleus (Gy) | Absorbed Dose to HER2 Cell Nucleus (Gy) |
|---|---|---|
| 0 | 0 | 0 |
| 1 | 1.3 | 0.65 |
| 5 | 6.5 | 3.3 |
| 10 | 13 | 6.5 |
| 20 | 26 | 13 |

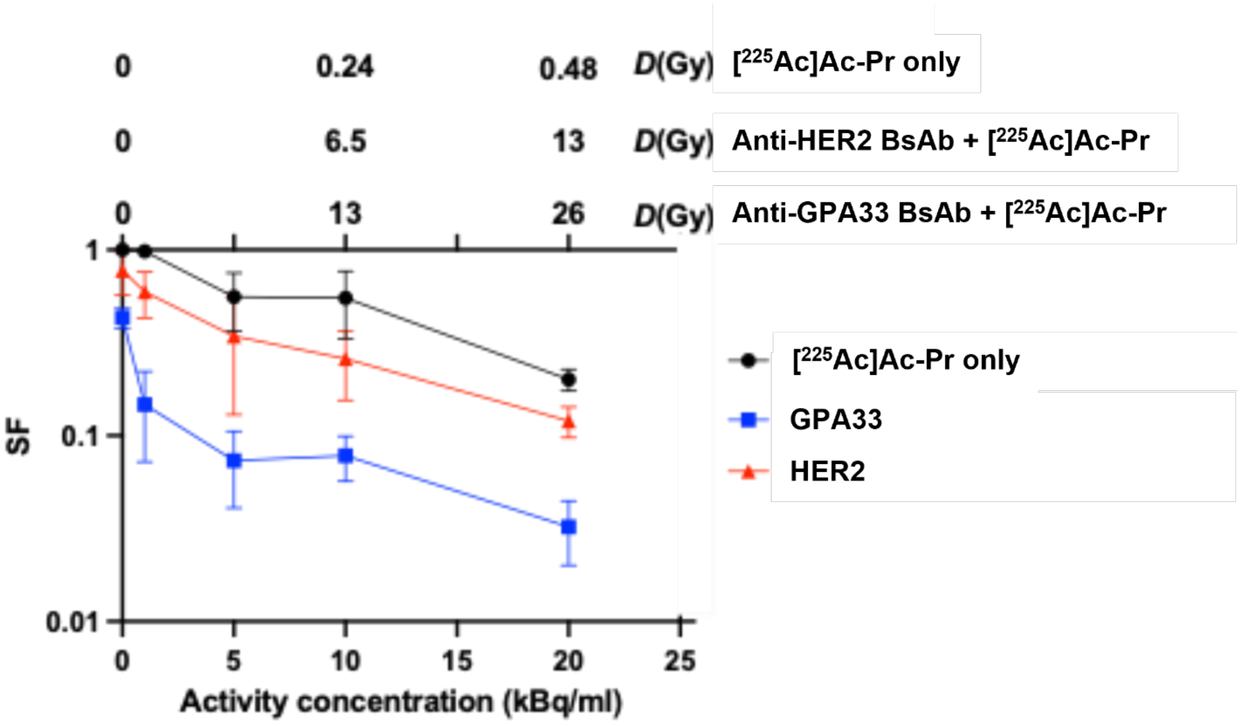


**Figure S3**: Surviving fraction of SW1222 cells as a function of activity concentration in the treatment medium (lower abscissa) and as a function of mean absorbed dose to the cell nucleus (upper abscissa). Note that the different absorbed dose scales for the three agents are due to differences in cellular uptake as a function of activity concentration.

*ROI analysis and quantification of autoradiography images*

The images resulting from the measurements using the iQID system were saved as 32-bit unsigned binary matrices with dimensions of 2448 x 2048 and an effective pixel size of 19.5 microns. The intensity in each pixel corresponds to the number of alpha particles detected, which directly correlates with the activity found in the sample (Figure 2B). Each structure in the image (kidney or tumor) was cropped out to create single images, with the structure centered in the new image. These one-structure images were analyzed using custom MATLAB scripts, performing different segmentations for tumors and kidney sections.

All imaged were first processed as follows:

1. Image Preprocessing and Smoothing
   Each cropped image was first loaded and normalized to a range of 0-255 to standardize the pixel intensity values. Gaussian smoothing was then applied to each image using a sigma value of 9.0. This smoothing step helped reduce noise and enhance the anatomical structures of interest.
2. Boundary Detection and Mask Creation
   The smoothed images were further processed to apply additional Gaussian blurring and perform thresholding using Otsu's method to convert the image into a binary format. Contours within the binary image were identified, and these contours were drawn and filled to create a macrostructure representing the isolated regions. The outermost boundary of this macrostructure was determined by evaluating each contour's area and

proximity to the image center, selecting the largest and most centrally located contour. A mask was generated from this outer boundary to further isolate the region of interest.

Kidney-Specific Image Segmentation:
Each kidney image was segmented into cortex and medulla automatically after the process described above. The mask created was applied to the original image to isolate the region of interest, effectively removing background noise. This isolated region was then segmented into cortex and medulla regions. The segmentation was manually refined to ensure accurate identification of the cortex and medulla.

Quantitative Analysis and Visualization:
After segmentation, quantitative analysis was performed on the cortex and medulla regions as well as tumors. Mean, median, and total intensities were calculated for each region. Ratios of each of these metrics for the tumor sections were taken to the respective ones for medulla, cortex, and total kidney for each animal.

*Pathology and IHC*
Mice were euthanized with carbon dioxide overdose, and terminal blood was collected via cardiac puncture for complete blood count and serum chemistry analyses. Following macroscopic examination, selected organs were fixed in 10% neutral-buffered formalin for 72 h, followed by decalcification of bones in a formic acid solution (Surgipath Decalcifier I, Leica Biosystems). Formalin-fixed tissues were then routinely processed in ethanol and xylene and embedded in paraffin in a Leica ASP6025 tissue processor. Paraffin blocks were sectioned at 5 microns and stained with hematoxylin and eosin (H&E). The following tissues were processed and examined: heart, thymus, lungs, liver, gallbladder, kidneys, pancreas, stomach, duodenum, jejunum, ileum, cecum, colon, lymph nodes (submandibular, mesenteric), salivary glands, skin (trunk and head), urinary bladder, uterus, cervix, vagina, ovaries, oviducts, adrenal glands, spleen, thyroid gland, esophagus, trachea, spinal cord, vertebrae, sternum, femur, tibia, stifle join, skeletal muscle, nerves, skull, nasal cavity, oral cavity, teeth, ears, eyes, pituitary gland, brain, and additional lesions, if present (e.g., xenograft).
HER2 IHC was performed by Center for Translational Pathology at New York-Presbyterian/Weill Cornell Medicine using a recombinant Anti-ErbB2 / HER2 antibody [SP3] (ab16662, Abcam). GPA33 IHC was performed according to O'Donoghue, et al. (1).

## Results

### *Uptake and Internalization*

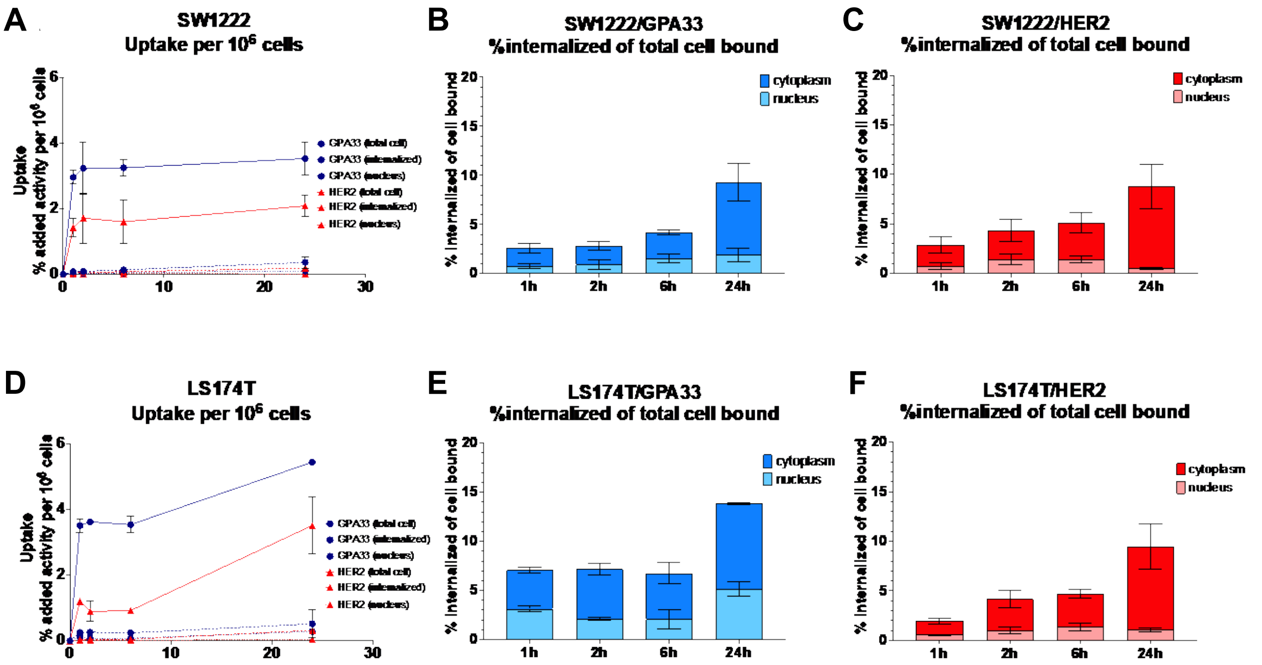


**Figure S4:** Uptake and internalization data in SW1222 (A-C) and LS174T (D-F) colorectal cancer cell lines. Cells were incubated with 64 nM of anti-GPA33 (blue) or anti-HER2 (red) BsAb for 1 hour prior to addition of 22 nM of [$^{111}$In]In-Pr. Internalized fraction was separated into cytoplasm and nucleus-associated activity (B, C, E, F).

**Table S3:** AUC analysis of uptake and internalization data showing absolute AUC values for total-cell bound, internalized and nucleus-associated activity. Data is based on the time activity graphs shown in Figure S4. *indicated statistically significant difference, $p < 0.05$, between GPA33 and HER2 targeting).

| AUC analysis | SW1222 | | LS174T | |
|---|---|---|---|---|
| | **GPA33** | **HER2** | **GPA33** | **HER2** |
| Total cell-bound (absolute AUC value) | 78.5 ± 5.3* | 42.1 ± 7.0 | 100.4 ± 2.4* | 45.1 ± 7.9 |
| Internalized activity (absolute AUC value) | 5.1 ± 1.6 | 2.7 ± 0.3 | 8.1 ± 3.8 | 3.44 ± 0.09 |
| Nucleus-associated activity (absolute AUC value) | 1.5 ± 0.8 | 0.39 ± 0.06 | 3.6 ± 0.5* | 0.50 ± 0.08 |
| % internalized of total bound | 6.45 % | 6.45 % | 8.11 % | 7.62 % |
| % nucleus-associated of total bound | 1.91 % | 0.93 % | 3.6 % | 1.1 % |
| % nucleus associated of internalized | 29.4 % | 14.4 % | 44.4 % | 14.5 % |

### *[$^{225}$Ac]Ac-Pr dosing and biodistribution*

The absolute tumor uptake (pmol/g tumor) of GPA33-pretargeted [$^{225}$Ac]Ac-Pr in SW1222 xenografts increased with injected dose indicating that even at 10000 pmol dose not all C825 binding sites are saturated. Tumor-to-blood ratio increased with increasing dose of [$^{225}$Ac]Ac-Pr whereas the tumor-to-

kidney ratio peaked between the 350 and 700 pmol dose with the tumor kidney ratio at 350 pmol (16 ± 6) being significantly higher than the tumor-to-kidney ratio at 10000 pmol (4 ± 1).

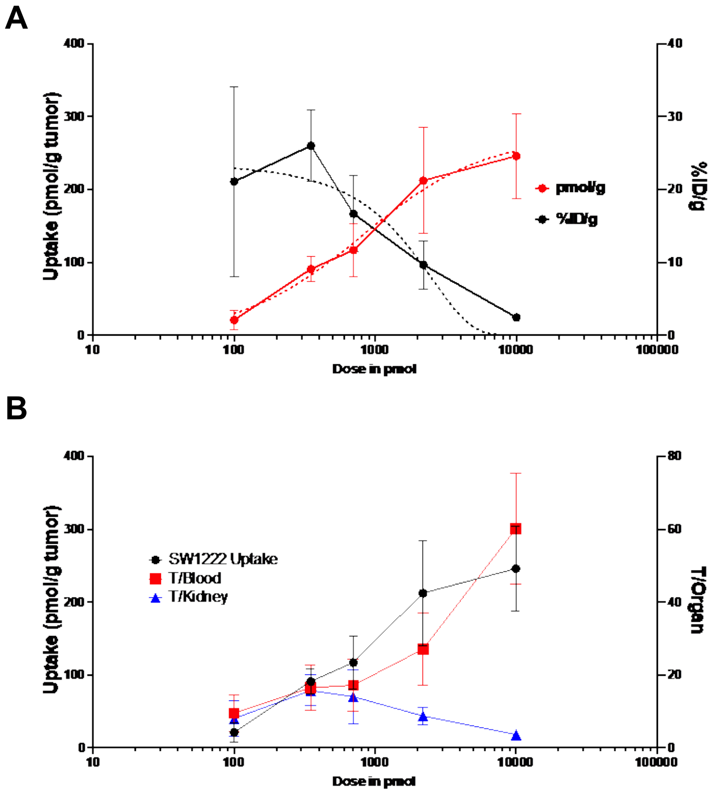


**Figure S5:** GPA33 targeting in SW1222 xenografts. (A) Dose-dependent uptake of [$^{225}$Ac]Ac-Pr in SW1222 xenografts at 24 h pi. Data presented as %ID/g (black line, right y-axis) and pmol/g of tumor (red line, left y-axis). (B) [$^{225}$Ac]Ac-Pr uptake in SW1222 xenografts (pmol/g tumor), and tumor-to-kidney (T/Kidney, blue) and tumor-to-blood (T/Blood, red) ratios at different radiohapten doses (24 h pi). [$^{225}$Ac]Ac-Pr was administered after anti-huGPA33 BsAb according to the described DOTA-PRIT regimen.

**Table S4:** Biodistribution of [$^{225}$Ac]Ac-Pr at different doses in SW1222 xenograft bearing mice injected with anti-GPA33 $^{225}$Ac-DOTA-PRIT over time. Data presented as average %ID/g ± SD (*n* = 4-5 mice/group).

| %ID/g | 100 pmol | 350 pmol | 700 pmol | 2190 pmol | 10000 pmol |
|---|---|---|---|---|---|
| **Blood** | 2.7± 0.7$^{b,c,d,e}$ | 1.8 ± 0.3$^{a,d,e}$ | 1.1 ± 0.4$^{a,e}$ | 0.4 ± 0.1$^{a,b}$ | 0.05 ± 0.02$^{a,b,c}$ |
| **SW1222** | 21 ± 13$^{e}$ | 26 ± 5$^{d,e}$ | 17 ± 5 | 10 ± 3$^{b}$ | 2.5 ± 0.6$^{a,b}$ |
| **Heart** | 1.7 ±0.3$^{b,c,d,e}$ | 1.2 ± 0.2$^{a,d,e}$ | 1.0 ± 0.3$^{a,d,e}$ | 0.55 ± 0.07$^{a,b,c}$ | 0.30 ± 0.05$^{a,b,c}$ |
| **Lungs** | 1.7 ± 0.7$^{c,d,e}$ | 1.3 ± 0.1$^{d,e}$ | 1.0 ± 0.2$^{a,e}$ | 0.42 ± 0.07$^{a,b}$ | 0.16 ± 0.01$^{a,b,c}$ |
| **Liver** | 9 ± 2$^{e}$ | 8.0 ± 0.6 | 8 ± 1 | 7 ± 2 | 6 ± 1$^{a}$ |
| **Spleen** | 2.4 ± 0.1$^{b,c,d,e}$ | 1.6 ± 0.2$^{a,c,d,e}$ | 1.0 ± 0.3$^{a,b,d,e}$ | 0.59 ± 0.03$^{a,b,c}$ | 0.36 ± 0.04$^{a,b,c}$ |
| **Stomach** | 1.09 ± 0.5$^{b,c,d,e}$ | 0.5 ± 0.1$^{a}$ | 0.50 ± 0.10$^{a}$ | 0.32 ± 0.05$^{a}$ | 0.29 ± 0.08$^{a}$ |
| **Small Intestine** | 1.0 ± 0.1$^{b,c,d,e}$ | 0.7 ±0.1$^{a,d,e}$ | 0.58 ± 0.07$^{a}$ | 0.35 ± 0.08$^{a,b}$ | 0.22 ± 0.04$^{a,b}$ |
| **Large Intestine** | 0.8 ± 0.3$^{c,d,e}$ | 0.5 ± 0.1 | 0.5 ± 0.2$^{a,d,e}$ | 0.32 ± 0.07$^{a,c}$ | 0.2 ± 0.1$^{a,c}$ |
| **Kidney** | 2.2 ± 0.5$^{c,d}$ | 1.7 ± 0.4$^{e}$ | 1.3 ± 0.4$^{a}$ | 1.1 ± 0.2$^{a}$ | 0.8 ± 0.2$^{b}$ |
| **Muscle** | 0.5 ± 0.3 | 0.4 ± 0.1 | 0.23 ± 0.07 | 0.2 ± 0.2 | 0.2 ± 0.2 |
| **Bone** | 2.8 ± 0.5$^{e}$ | 2.7 ± 0.8$^{e}$ | 2.1 ± 0.6 | 1.7 ± 0.6 | 1.5 ± 0.5$^{a,b}$ |
| **Tail** | 1.8 ± 0.2 | 2 ± 1 | 2 ± 2 | 1.0 ± 0.2 | 0.73 ± 0.10 |

Statistically significant difference between group indicated by $^{a}$ vs 100 pmol, $^{b}$ vs 350 pmol, $^{c}$ vs 700 pmol, $^{d}$ vs 2100 pmol, $^{e}$ vs 10000 pmol

**Table S5:** Tumor-to-tissue ratios. Biodistribution of [$^{225}$Ac]Ac-Pr at different doses in SW1222 xenograft bearing mice injected with anti-GPA33 $^{225}$Ac-DOTA-PRIT over time. Data presented as average ± SD (*n* = 4-5 mice/group).

| Tumor/Tissue Ratio | 100 pmol | 350 pmol | 700 pmol | 2190 pmol | 10000 pmol |
|---|---|---|---|---|---|
| **Blood** | 8 ± 5$^{d,e}$ | 16 ± 4$^{e}$ | 17 ± 7$^{e}$ | 27 ± 10$^{a,e}$ | 60 ± 15$^{a,b,c,d}$ |
| **Heart** | 13 ± 9$^{d}$ | 22 ± 5 | 17 ± 5 | 18 ± 8$^{a}$ | 9 ± 3 |
| **Lungs** | 12 ± 6$^{d}$ | 20 ± 6 | 17 ± 5 | 22 ± 5$^{a}$ | 15± 3 |
| **Liver** | 2 ± 1$^{f}$ | 3.4 ± 0.8$^{d,e}$ | 2.2 ± 0.6$^{e}$ | 1.6 ± 0.8$^{b}$ | 0.4 ± 0.1$^{a,b,c}$ |
| **Spleen** | 8 ± 6 | 17 ± 3 | 18 ± 8 | 16 ± 6 | 7 ± 2 |
| **Stomach** | 24 ± 21$^{b}$ | 57 ± 15$^{a,e}$ | 36± 16 | 31 ± 10 | 10 ± 4$^{b}$ |
| **Small Intestine** | 21 ± 14 | 40 ± 11$^{e}$ | 30 ± 10 | 27 ± 6 | 11± 3$^{b}$ |
| **Large Intestine** | 27 ± 15 | 53 ± 6$^{e}$ | 44 ± 32 | 31 ± 12 | 10 ± 8$^{b}$ |
| **Kidney** | 10 ± 5 | 16 ± 6 | 14 ± 7$^{e}$ | 9 ± 2 | 4 ± 1$^{c}$ |
| **Muscle** | 44 ± 21 | 81 ± 31$^{e}$ | 77 ± 30 | 83± 72 | 30 ± 18$^{b}$ |
| **Bone** | 8 ± 5 | 12 ± 4$^{e}$ | 8 ± 4 | 6 ± 3 | 2 ± 0.7$^{b}$ |
| **Tail** | 12 ± 7 | 17 ± 9$^{e}$ | 10 ± 5 | 10 ± 3 | 4 ± 1$^{b}$ |

Statistically significant difference between group indicated by $^{a}$ vs 100 pmol, $^{b}$ vs 350 pmol, $^{c}$ vs 700 pmol, $^{d}$ vs 2100 pmol, $^{e}$ vs 10000 pmol

*Biodistribution of [$^{225}$Ac]Ac-Pr in mice with SW1222 xenografts*

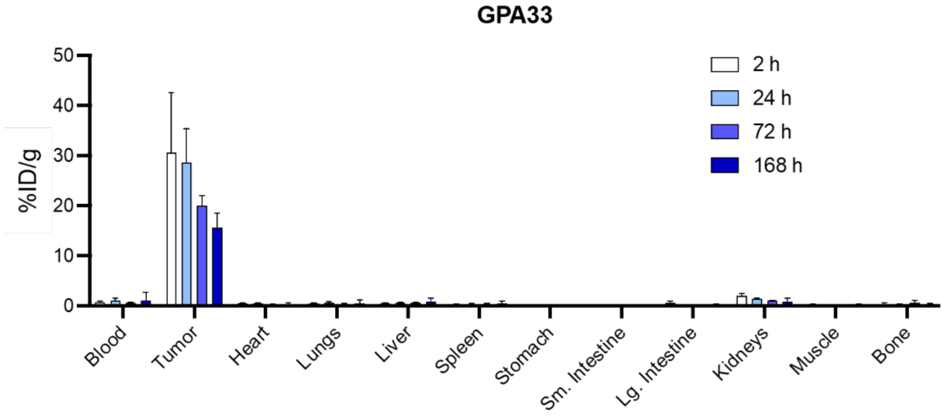


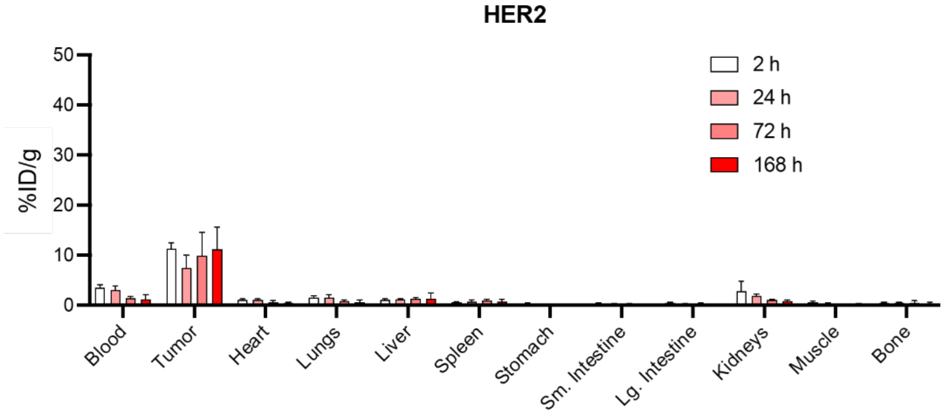


**Figure S6:** Biodistribution of [$^{225}$Ac]Ac-Pr (700 pmol) in SW1222 xenograft bearing mice injected with anti-GPA33 $^{225}$Ac-DOTA-PRIT (top) anti-HER2 $^{225}$Ac-DOTA-PRIT (bottom) over time. Data presented as average %ID/g ± SD (*n* = 4-5 mice/group).

**Table S6:** Biodistribution of [$^{225}$Ac]Ac-Pr (700 pmol) in SW1222 xenograft bearing mice injected with anti-GPA33 $^{225}$Ac-DOTA-PRIT over time. Data presented as average %ID/g ± SD (*n* = 4-5 mice/group).

| GPA33, %ID/g | 2 h | 24 h | 72 h | 168 h |
|---|---|---|---|---|
| **Blood** | 0.8± 0.2[*] | 1.1 ± 0.4[*] | 0.7 ± 0.1[*] | 1 ± 1 |
| **Tumor** | 31 ± 12[d,] | 29 ± 7[*] | 20 ± 2[*] | 16 ± 3[a] |
| **Heart** | 0.4 ± 0.1[*] | 0.5 ± 0.1[*] | 0.28 ± 0.06[*] | 0.3 ± 0.3 |
| **Lungs** | 0.51 ± 0.06[*] | 0.6 ± 0.2[*] | 0.39 ± 0.09[*] | 0.5 ± 0.7 |
| **Liver** | 0.51 ± 0.09[*] | 0.6 ± 0.1[*] | 0.66 ± 0.06[*] | 0.9 ± 0.7 |
| **Spleen** | 0.29 ± 0.06[*] | 0.38 ± 0.07[*] | 0.41 ± 0.09[*] | 0.4 ± 0.4 |
| **Stomach** | 0.13 ± 0.06 | 0.09 ± 0.04[*] | 0.06 ± 0.02[*] | 0.08 ± 0.07 |
| **Sm. Intestine** | 0.14 ± 0.04[*] | 0.1 ± 0.03[*] | 0.09 ± 0.03[*] | 0.1 ± 0.1 |
| **Lg. Intestine** | 0.7 ± 0.3[b,c,d] | 0.19 ± 0.09[a] | 0.15 ± 0.07[a] | 0.1 ± 0.2[a] |
| **Kidneys** | 2.1 ± 0.4[b,c,d] | 1.4 ± 0.2[a,*] | 1.0 ± 0.1[a] | 0.8 ± 0.8[a] |
| **Muscle** | 0.3 ± 0.1 | 0.16 ± 0.04[*] | 0.14 ± 0.06 | 0.2 ± 0.2 |
| **Bone** | 0.3 ± 0.3 | 0.2 ± 0.1[*] | 0.7 ± 0.4 | 0.3 ± 0.2 |

Statistical significance indicated by [a]vs 2h, [b] vs 24 h, [c]vs 72 h, [d]vs 168 h (One-way ANOVA with post-hoc t-test adjusted for multiple comparisons with Tukey) *vs HER2 at same timepoint (simple t-test)

**Table S7:** Biodistribution of [$^{225}$Ac]Ac-Pr (700 pmol) in SW1222 xenograft bearing mice injected with anti-HER2 $^{225}$Ac-DOTA-PRIT over time. Data presented as average %ID/g ± SD (*n* = 4-5 mice/group).

| HER2, %ID/g | 2 h | 24 h | 72 h | 168 h |
|---|---|---|---|---|
| **Blood** | 3.5 ± 0.6$^{c,d,*}$ | 3.1 ± 0.8$^{c,d,*}$ | 1.4 ± 0.4$^{a,b,*}$ | 1.2 ± 0.9$^{a,b}$ |
| **Tumor** | 11 ± 1 | 7 ± 3$^{*}$ | 10 ± 5$^{*}$ | 11 ± 4 |
| **Heart** | 1.0 ± 0.2$^{d,*}$ | 1.0 ± 0.3$^{d,*}$ | 0.6 ± 0.3$^{*}$ | 0.4 ± 0.2$^{a,b}$ |
| **Lungs** | 1.4 ± 0.2$^{d,*}$ | 1.6± 0.6$^{d,*}$ | 0.9 ± 0.3$^{*}$ | 0.6± 0.4$^{a,b}$ |
| **Liver** | 1.0 ± 0.2$^{*}$ | 1.1 ± 0.2$^{*}$ | 1.3 ± 0.2$^{*}$ | 1 ± 1 |
| **Spleen** | 0.5 ± 0.1$^{*}$ | 0.7 ± 0.3$^{*}$ | 0.9 ± 0.3$^{*}$ | 0.8 ± 0.4 |
| **Stomach** | 0.6 ± 0.2 | 0.18 ± 0.06$^{*}$ | 0.16 ± 0.03$^{*}$ | 0.12 ± 0.06 |
| **Sm. Intestine** | 0.36 ± 0.09$^{d,*}$ | 0.28 ± 0.05$^{*}$ | 0.22 ± 0.08$^{*}$ | 0.20 ± 0.09$^{a}$ |
| **Lg. Intestine** | 0.5 ± 0.2$^{d}$ | 0.29 ± 0.05 | 0.3 ± 0.2 | 0.15 ± 0.03$^{a}$ |
| **Kidneys** | 1.9 ± 3$^{c,d}$ | 1.9 ± 0.3$^{c,d*}$ | 1.0 ± 0.2$^{a,b}$ | 0.8 ± 0.2$^{a,b}$ |
| **Muscle** | 0.5 ± 0.1$^{c,d}$ | 0.3 ± 0.1$^{*}$ | 0.19 ± 0.02$^{a}$ | 0.2 ± 0.1$^{a}$ |
| **Bone** | 0.5 ± 0.2 | 0.49 ± 0.07$^{*}$ | 0.5 ± 0.4 | 0.3 ± 0.3 |

Statistical significance indicated by $^{a}$vs 2h, $^{b}$ vs 24 h, $^{c}$vs 72 h, $^{d}$vs 168 h (One-way ANOVA with post-hoc t-test adjusted for multiple comparisons with Tukey), *vs GPA33 at same timepoint (simple t-test)

## *Quantification of iQID autoradiography images*

**Table S8:** ROI intensities (number of alpha-particle counts in ROI) for iQID image acquisitions. Each row represents data from one animal.

| Target and time point | **Tumor (SW1222)** | | | **Kidney (Cortex)** | | | **Kidney (Medulla)** | | |
|---|---|---|---|---|---|---|---|---|---|
| | Mean Intensity | Median Intensity | Total Intensity | Mean Intensity | Median Intensity | Total Intensity | Mean Intensity | Median Intensity | Total Intensity |
| **GPA33 2 h pi** | 9.1 | 10.6 | 680,542 | 0.60 | 0.03 | 35,640 | 0.29 | 0.02 | 9,950 |
| | 4.5 | 4.0 | 261,312 | 0.56 | 0.03 | 34,276 | 0.18 | 0.02 | 5,981 |
| **GPA33 24 h pi** | 5.0 | 5.3 | 185.996 | 0.42 | 0.02 | 24,900 | 0.14 | 0.02 | 2,735 |
| | 3.8 | 2.6 | 132,878 | 0.48 | 0.02 | 18,655 | 0.14 | 0.02 | 4,068 |
| **HER2 2 h pi** | 1.2 | 1.1 | 53.677 | 0.79 | 0.05 | 33,969 | 0.44 | 0.04 | 22,159 |
| **HER2 24 h pi** | 1.3 | 1.2 | 58,017 | 0.66 | 0.03 | 32,330 | 0.39 | 0.02 | 15,356 |
| | 1.3 | 1.2 | 57,995 | 0.60 | 0.03 | 38,228 | 0.35 | 0.02 | 12,533 |

## $^{225}$Ac-DOTA-PRIT therapy in a SW1222 flank model

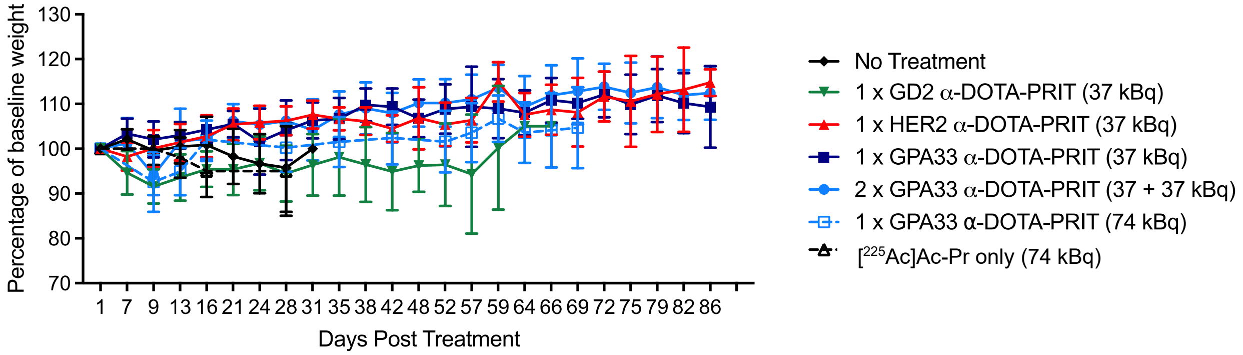


**Figure S7:** Weight curves for mice with SW1222 xenograft treated with $^{225}$Ac-DOTA-PRIT. Bodyweight was monitored weekly and is displayed as percent of bodyweight at baseline.

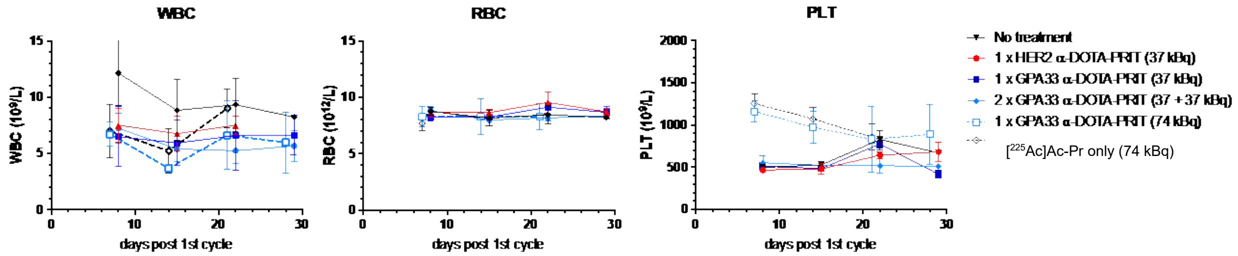


**Figure S8:** Hematological analysis from SW1222 xenograft bearing mice treated with $^{225}$Ac-DOTA-PRIT showing white blood cell count (left), red blood cell count (middle) and platelet counts (right). Blood was sampled once a week over the course of 4 weeks post [$^{225}$Ac]Ac-Pr administration.

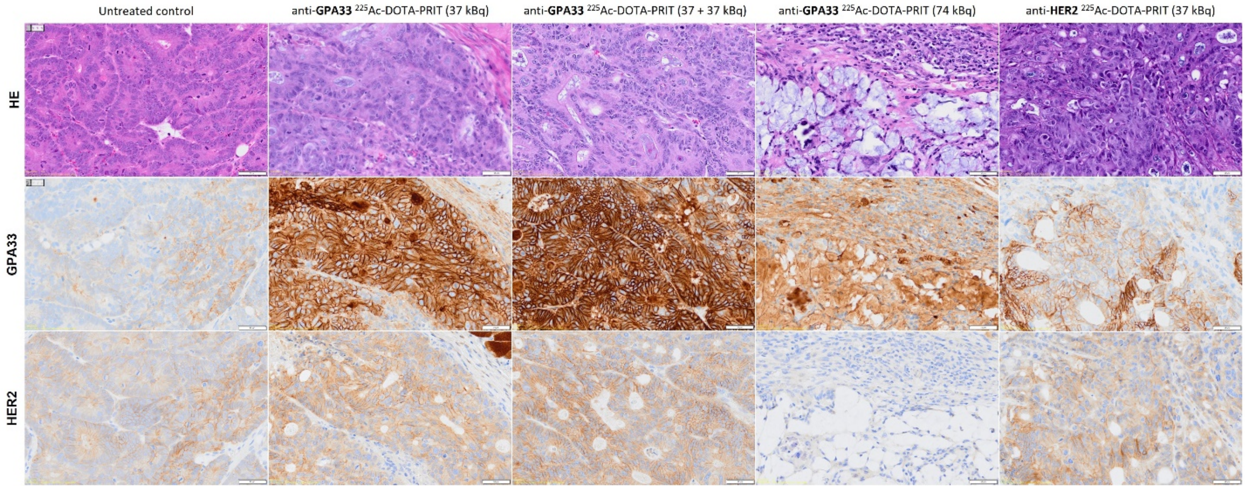


**Figure S9:** Hematoxylin and eosin (HE [top]), GPA33 IHC (middle), HER2 IHC (bottom) staining of recurred SW1222 xenografts after treatment $^{225}$Ac-DOTA-PRIT. First column, SW1222 xenograft of untreated control group: mild GPA33 expression; mild to moderate HER2 expression. Second column, SW1222 xenograft of 1 x anti-**GPA33** $^{225}$Ac-DOTA-PRIT (37 kBq) group (2/10 animals alive at 124 d and 1 residual tumor): markedly increased GPA33 expression in neoplastic cells; moderate HER2 expression. Third column, 2 x anti-**GPA33** $^{225}$Ac-DOTA-PRIT (37 + 37 kBq) group (5/10 animals alive at 124 d and 3 residual tumors): markedly increased GPA33 expression in neoplastic cells; moderate HER2 expression. Fourth column, 1 x anti-**GPA33** $^{225}$Ac-DOTA-PRIT (74 kBq) group (3/10 animals alive at 124 d and no residual tumor): nonspecific background staining in areas of necrosis, mucin, and chronic inflammation, but no neoplastic cells identified. Fifth column, 1 x anti-**HER2** $^{225}$Ac-DOTA-PRIT (37 kBq) group, (2/10 animals alive at 124 d and 1 residual tumor): mild to moderate GPA33 expression in neoplastic cells; moderate HER2 expression. Chromogen: DAB. Counterstaining: Hematoxylin. Scale bar: 50 µm.

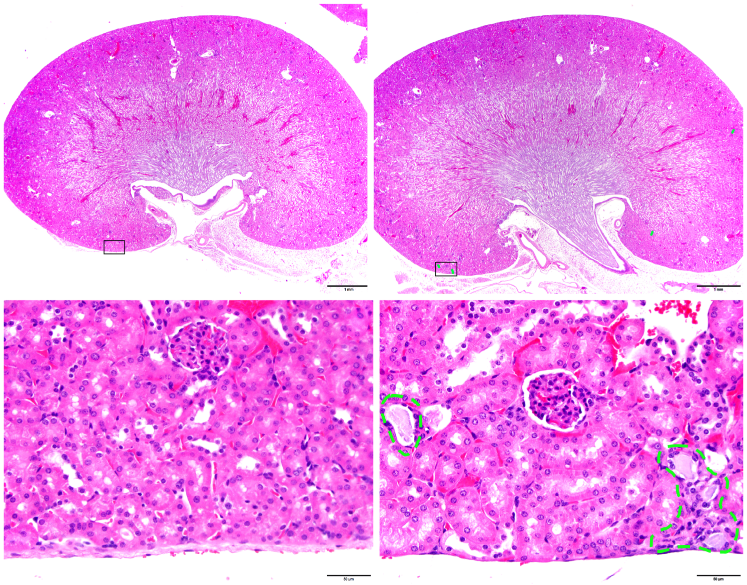

**Figure S10:** Representative kidney images of mice treated with anti-GPA33 $^{225}$Ac-DOTA-PRIT. Normal kidney on the left (control group) and kidney affected by therapy on the right (2 x 37 kBq anti-GPA33 $^{225}$Ac-DOTA-PRIT group). While these changes were probably caused by the treatment, they did not appear to affect renal function or general health of the animals, thus they were not considered adverse. Scalebar: 1 mm (top), 50 µm (bottom).

**Table S9:** Absorbed doses to tissues and therapeutic indices based on serial biodistribution of anti-GPA33 and anti-HER2 $^{225}$Ac-DOTA-PRIT in SW1222 xenografted mice. Dose shown as Gy/MBq. Estimation does not include cross-dose between organs, total local absorption assumed.

| Tissue | Tissue doses (Gy/MBq) | | | | | | | |
|---|---|---|---|---|---|---|---|---|
| | **GPA33** | | | | **HER2** | | | |
| | $^{225}$Ac + first three daughters | $^{225}$Ac + all daughters | $^{225}$Ac + first three daughters (RBE=5) | $^{225}$Ac + all daughters (RBE=5) | $^{225}$Ac + first three daughters | $^{225}$Ac + all daughters | $^{225}$Ac + first three daughters (RBE=5) | $^{225}$Ac + all daughters (RBE=5) |
| Blood | 39.007 | 58.869 | 195.035 | 294.345 | 56.527 | 85.311 | 282.635 | 426.555 |
| **Tumor** | **692.360** | **1044.915** | **3461.8** | **5224.575** | **407.969** | **615.710** | **2039.845** | **3078.55** |
| Heart | 11.442 | 17.269 | 57.21 | 86.345 | 20.432 | 30.837 | 102.16 | 154.185 |
| Lungs | 19.268 | 29.079 | 96.34 | 145.395 | 30.434 | 45.932 | 152.17 | 229.66 |
| Liver | 30.428 | 45.922 | 152.14 | 229.61 | 49.612 | 74.875 | 248.06 | 374.375 |
| Spleen | 15.752 | 23.773 | 78.76 | 118.865 | 29.521 | 44.553 | 147.605 | 222.765 |
| Stomach | 3.035 | 4.581 | 15.175 | 22.905 | 5.198 | 7.845 | 25.99 | 39.225 |
| Sm. Intestine | 4.674 | 7.055 | 23.37 | 35.275 | 8.181 | 12.347 | 40.905 | 61.735 |
| Lg. Intestine | 6.205 | 9.365 | 31.025 | 46.825 | 7.335 | 11.070 | 36.675 | 55.35 |
| Kidneys | 36.182 | 54.606 | 180.91 | 273.03 | 37.599 | 56.744 | 187.995 | 283.72 |
| Muscle | 6.037 | 9.111 | 30.185 | 45.555 | 9.087 | 13.715 | 45.435 | 68.575 |
| Bone | 12.990 | 19.605 | 64.95 | 98.025 | 13.651 | 20.602 | 68.255 | 103.01 |

**Table S10.** Statistical comparison of MS from *in vivo* therapy in SW1222 flank model. Resulting p-values from with log-rank (Mantel-Cox) test.

| p-values | Untreated control | 1 x GD2 α-DOTA-PRIT (37 kBq) | 1 x HER2 α-DOTA-PRIT (37 kBq) | 1 x GPA33 α-DOTA-PRIT (37 kBq) | 2 x GPA33 α-DOTA-PRIT (37 + 37 kBq) | 1 x GPA33 α-DOTA-PRIT (74 kBq) |
|---|---|---|---|---|---|---|
| **Untreated control** | | 0.0364 | 0.0038 | 0.0006 | <0.0001 | <0.0001 |
| **1 x GD2 α-DOTA-PRIT (37 kBq)** | 0.0364 | | 0.0024 | <0.0001 | <0.0001 | <0.0001 |
| **1 x HER2 α-DOTA-PRIT (37 kBq)** | 0.0038 | 0.0024 | | 0.6124 | 0.0043 | 0.0896 |
| **1 x GPA33 α-DOTA-PRIT (37 kBq)** | 0.0006 | <0.0001 | 0.6124 | | 0.0011 | 0.0961 |
| **2 x GPA33 α-DOTA-PRIT (37 + 37 kBq)** | <0.0001 | <0.0001 | 0.0043 | 0.0011 | | 0.1390 |
| **1 x GPA33 α-DOTA-PRIT (74 kBq)** | <0.0001 | <0.0001 | 0.0896 | 0.0961 | 0.1390 | |

**Table S11:** Histologic scoring of renal degenerative tubulointerstitial changes (based on (2))

| **Tubulointerstitial Features** | **2 x GPA33 α-DOTA-PRIT (37 + 37 kBq)** | | | | | **1 x GPA33 α-DOTA-PRIT (37 kBq)** | **1 x HER2 α-DOTA-PRIT (37 kBq)** | | **1 x GPA33 α-DOTA-PRIT (74 kBq)** | | | **Anti CD33 [225Ac]Ac-mAb*** |
|---|---|---|---|---|---|---|---|---|---|---|---|---|
| Nuclear changes (% of cells) | <1% Karyomegaly | 0 | <1% Karyomegaly | 0 | 0 | <1% Karyomegaly | <1% Karyomegaly <1% Karyorrhexis | <1% Karyomegaly <1% Karyorrhexis | <1% Karyomegaly <1% Karyorrhexis | <1% Karyomegaly | <1% Karyomegaly | Moderate, focal karyorrhexis |
| Cytoplasmic vacuolation (% of cells) | <1% | <1% | <1% | <1% | <1% | <1% | <1% | <1% | <1% | <1% | <1% | >50 |
| Tubulolysis with collapse (% of tubules) | 0 | 0 | 0 | 0 | 0 | 0 | 0 | 0 | 0 | 0 | 0 | >50 |
| Loss of brush border (% of tubules) | <1% | <1% | <1% | 0 | 0 | <1% | <1% | <1% | <1% | 0 | 0 | 70 |
| Atrophy (% of tubules) | <1% | 0 | <1% | 0 | 0 | <1% | <1% | <1% | <1% | 0 | 0 | --- |
| Shrinkage/simplification of tubules (% of tubules) | <1% | 0 | <1% | 0 | 0 | <1% | <1% | <1% | <1% | 0 | 0 | 50 |
| Tubular casts (% of tubules) | <1% | 0 | <1% | 0 | 0 | <1% | <1% | <1% | <1% | <1% | <1% | Rare |
| Interstitial inflammation | Minimal, multifocal lymphocytic infiltrates | Minimal, multifocal lymphocytic infiltrates | Minimal, multifocal lymphocytic infiltrates | 0 | 0 | Minimal, multifocal lymphocytic infiltrates | Minimal, multifocal lymphocytic infiltrates | Minimal, multifocal lymphocytic infiltrates | 0 | 0 | Minimal, focal lymphocytic infiltrates | --- |
| Interstitial fibrosis | 0 | 0 | 0 | 0 | 0 | 0 | 0 | 0 | 0 | 0 | 0 | --- |
| Medullary tubules | Normal | Normal | Normal | Normal | Normal | Normal | Normal | Normal | Normal | Normal | Normal | Normal |

*From Jaggi et al. (2) Damage at 20 weeks.

*PDX Therapy*

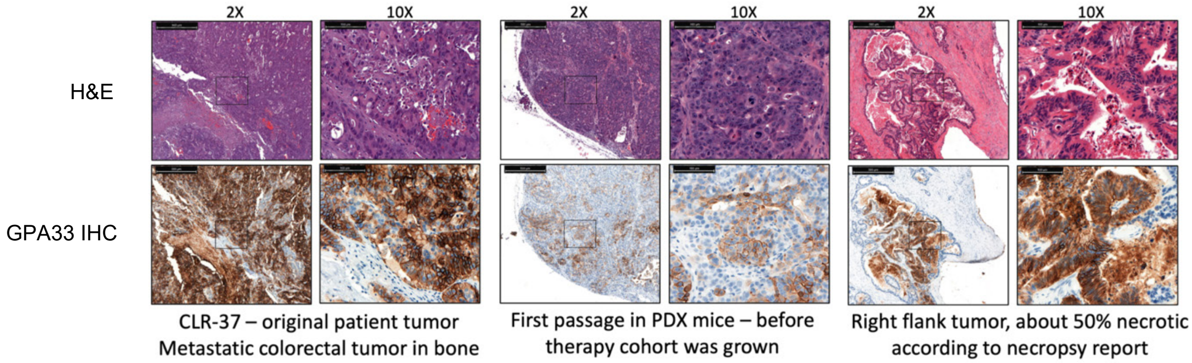


**Figure S11:** IHC of CLR37 confirming GPA33 expression in the original patient tumor (left), PDX after first passage in mice and a flank tumor before implantation (middle) and a flank tumor of a therapy control mouse (right).

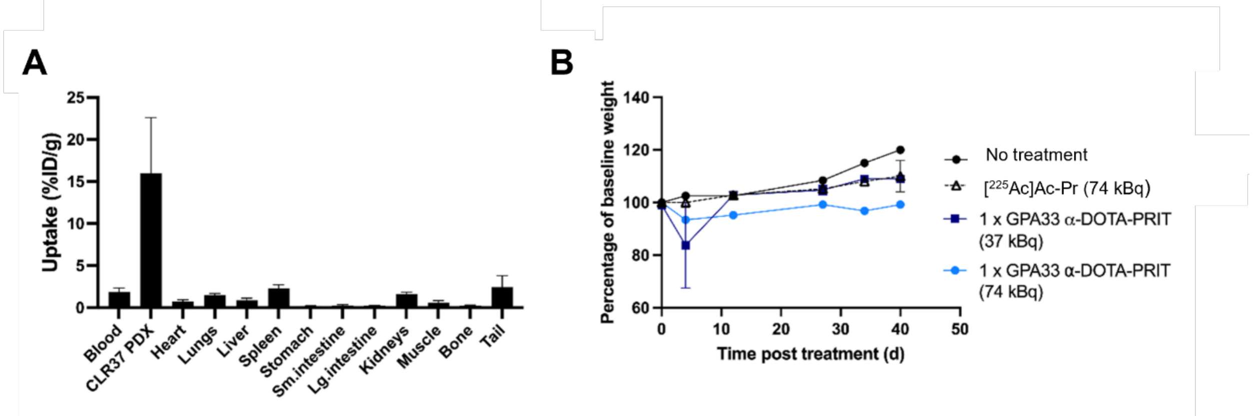


**Figure S12:** Biodistribution and experimental therapy in mice with CLR37 PDX. (A) Targeting of GPA33 in GPA33(+) CLR37 PDX $^{225}$Ac-DOTA-PRIT 24 h pi ($n$ = 4, average ± SD). Mice were pre-injected with anti-GPA33 BsAb, clearing agent, and [$^{225}$Ac]Ac-Pr according to the DOTA-PRIT regimen. (B) Weight changes.

## References


1. O'Donoghue JA, Smith-Jones PM, Humm JL, Ruan S, Pryma DA, Jungbluth AA*, et al.* 124I-huA33 antibody uptake is driven by A33 antigen concentration in tissues from colorectal cancer patients imaged by immuno-PET. J Nucl Med 2011; 52: 1878-85
2. Jaggi JS, Seshan SV, McDevitt MR, LaPerle K, Sgouros G, Scheinberg DA. Renal tubulointerstitial changes after internal irradiation with alpha-particle-emitting actinium daughters. J Am Soc Nephrol 2005; 16: 2677-89
